# Influence of Cellulose Nanofibers on the Behavior of Pickering Emulsions. Part 1. Microscopy and Startup Flow Test

**DOI:** 10.3390/ma15238285

**Published:** 2022-11-22

**Authors:** Shu-Ming Cui, Saud Hashmi, Wen-Qiang Li, Stephan Handschuh-Wang, Cheng-Tian Zhu, Shi-Chang Wang, Pian-Pian Yang, Yan-Fei Huang, Guang-Ming Zhu, Florian J. Stadler

**Affiliations:** 1 Shenzhen Key Laboratory of Polymer Science and Technology, Guangdong Research Center for Interfacial Engineering of Functional Materials, Nanshan District Key Laboratory for Biopolymers and Safety Evaluation, College of Materials Science and Engineering, Shenzhen University, Shenzhen 518055, China; 2 South China Advanced Institute for Soft Matter Science and Technology, School of Emergent Soft Matter, South China University of Technology, Guangzhou 510640, China; 3 Department of Polymer & Petrochemical Engineering, NED University of Engineering & Technology, Karachi 75270, Sindh, Pakistan; 4 College of Chemistry and Environmental Engineering, Shenzhen University, Shenzhen 518060, China; 5 College of Management, Shenzhen University, Shenzhen 518055, China

**Keywords:** cellulose nanofibers, CNF networks, physical entanglement, droplets clusters, startup flow

## Abstract

The dispersibility of flexible polymer chains present at the emulsion’s interface between the dispersed and continuous phase has obvious effects on rheology and dielectric properties of the whole emulsion. Cellulose nanofiber (CNF)-based Pickering emulsions are good systems to research these properties with respect to their microscopic phase structure, dielectric, and rheological properties by using CNF as a water-dispersible Pickering emulsifier, liquid paraffin as an oil phase, and didodecyldimethylammonium bromide (DDAB) as a cationic auxiliary surfactant. The CNF and DDAB contents were systematically varied while the water-to-paraffin oil ratio was kept constant to discern the influence of the Pickering emulsifiers. Polarized optical microscopic images reveal that the droplets tend to shrink at higher CNF content but grow bigger when increasing the DDAB content, which is proved by fluorescence analysis of the CNF dispersibility with varying DDAB content. The dielectric damping exhibits a minimum, whose value decreases with increasing DDAB and CNF content. Increasing the DDAB content promotes the solubilization of CNF in the aqueous phase, which will increase the overall viscosity and yield points. Similarly, a higher CNF content leads to a higher viscosity and yield point, but at high DDAB contents, the viscosity function exhibits an S-shape at intermediate CNF contents. To evaluate the results further, they were compared with CNF dispersions (without oil phase), which showed a surfactant effect slightly on maximum stress but strongly on yield stress $\tau_{y}$, indicating that DDAB can promote the formation of a CNF network rather than the viscosity of the whole system. This paper provides information on how a systematical variation of the composition influences morphology and physico-chemical interactions as detected by broadband dielectric spectroscopy and rheological behavior.

## 1. Introduction

An emulsion is a dispersed system composed of two immiscible liquid phases, usually an oil and an aqueous phase with an emulsifier (surfactant or Pickering emulsifier) adsorbed at the interface. Besides those, also immiscible polymer blends are important emulsions, when both components are in the molten state. One attractive emulsifier is cellulose. Cellulose, the natural, environmental, and renewable material, consists of dehydrated glucose units. These glucose (hexose) units comprise abundant hydroxy and carboxyl moieties as hydrophilic groups and a polymer backbone and are widely applied as biocompatible emulsifiers and thickeners. Nanocellulose refers to ultra-fine cellulose. It is an amphiphilic organic polymer, with a diameter between 1~100 nm. Cellulose nanocrystal (CNC) [1,2,3], microfibrillated cellulose (MFC) [4,5,6], bacterial nanocellulose (BNC) [7,8,9,10], and cellulose nanofibers (CNF) [11] belong to the class of nanocellulose. In general, emulsions stabilized with CNF or CNC can behave in a gel-like manner because of strong and irreversible cellulose adsorption to the O/W (oil/water) interface [2], based on steric interfacial stabilization [12]. As spindle-like or rod-like particles, CNCs possess near-perfect crystallinity (ca. 90%), while CNF with micrometer-long entanglements contains both amorphous and crystalline domains [12,13,14]. CNC and CNF possess abundant hydroxyl groups that allow potential hydrogen bonds and surface modification, which the surface chemistry associates with pre-treatment and refinement approaches from specific cellulose sources [15,16]. In appearance, different aspect ratio celluloses impact the rheology and morphology of corresponding dispersion and emulsion. For dispersion formation by CNC and CNF, Du et al. [17] reported that CNC dispersion, containing needle-like crystals of smaller aspect ratio, exhibited elastic gel-like rheological properties, but at lower CNC concentration, a concentration dependence on viscosity was found, i.e., a liquid-like behavior. On the contrary, CNF dispersions with larger aspect ratios exhibited solid-like viscoelastic behavior and larger concentrations result in increasing viscosity and its plateau at low shear rates, which associate from the specific entangled network. For their stabilized emulsion, Li et al. [18] studied different CNC particles fabricated from pure CNF by hydrolyzation with sulfuric acid-for 1, 2, and 4 h. The viscosity curves of pure CNF emulsions present the obvious sharp decrease at about 10 s^−1^, however, the mixture of CNF and crystallized CNC did not show that anymore, which proved the crosslinked feature in the aqueous phase affected CNF flexible matrixes rather than the dense aggregation of rod-like CNC particles. Furthermore, Kalashnikova et al. [19] proved that long cellulose nanocrystals with densely entangled chains could form ultrastable emulsions and interconnected networks of low covered droplets (40%), compared with shorter nanocrystals that merely feature dense interfacial absorption rather than a role in connection among droplets. Physical entanglements of CNF and solid-like viscoelastic properties can be easily created in the aqueous phase due to the large aspect ratio and high flexibility of CNF, even at very low concentrations, and this sets them apart from short and rod-like CNC structures, which necessitate higher concentrations. In addition, CNF-based emulsions feature a certain shear-thinning behavior [17,18,20]. In terms of surface chemistry, CNF contains plentiful hydroxyl groups, which promotes their dispersion in aqueous solutions. However, the nature of the large aspect ratio, large surface area, and high interface energy drives CNF to self-assemble, while abundant hydroxyl groups promote chains dispersion. Thus, CNF clusters in dispersion are inhomogeneous without any chemical modification that improve compatibility and homogeneous dispersion within polymer matrixes to obtain a stronger network [21,22]. For instance, Tan et al. [21] reported that chemical crosslinks consisting of γ-aminopropyltriethoxysilane grafted to the CNF chain reduce the free hydroxyl groups, weaken the interaction, and improve the distribution by means of the formation of C−O−Si bond, with the modified CNF dispersion altering the shear thickening, resulting from the G′, G′′ vs. ω plotted curves with positive slope.

Pickering particles cannot maintain the interfacial force balance merely by intermolecular forces due to weak interfacial interactions associated with balanced contact angle between aqueous and oil phases, possibly resulting in droplet rupture in the course of rapid rheology profiles or long-term storage [23,24,25]. Hence, surfactants are often employed to overcome this issue. When a surfactant has a strong electrostatic attraction with the Pickering stabilizers and dispersed droplets, it promotes the formation of complex interfaces rather than desorption of the particles [26]. In other words, additional ionic surfactants can establish a bridge between particles and dispersed droplets through stronger ionic adsorption to enhance interfacial stability, which is essential for stabilizing emulsion systems [27]. For instance, Hong et al. [28] studied the effect of the addition of cetyl trimethyl ammonium bromide (CTAB) on the improvement of the interfacial attraction between clays and oil and the associated increase in the interfacial modulus. These effects are strong enough to provide a barrier against droplet coalescence. Similarly, Whitby et al. [29] concluded that the auxiliary surfactant (octadecylamine) is able to affect the aggregate’s state of Pickering particles (laponite) in water. Specifically, the surfactant affects the droplets’ size and prevents droplets’ coalescence in the emulsion by means of the synergistic and antagonistic interactions between laponite and octadecylamine [25]. In conclusion, the presence of auxiliary ionic surfactants improves the interfacial adsorption of stabilizers at the interface by stronger electrostatic connection. If this mechanism is not present, the interfacial modulus would decrease due to the stabilizers’ desorption against interface [28].

It is vital for this article to discuss the distribution of CNF chains at the interface and in the continuous phase in emulsion systems using changing CNF and auxiliary surfactant content. Polarized optical, electronic, and fluorescence microscopy and steady rheological studies are analyzed to assess the droplet’s size, CNF distribution, and bulk flow behavior.

Broadband dielectric spectroscopy (BDS) can study the structure and stability of emulsions and their response to alternating current (AC) fields, not only for dielectric properties affected by droplets and surfaces, but also abnormal states, such as phase separation, flocculation, and droplet coalescence [30]. BDS uses a frequency generator to create a sinusoidal electrical signal applied to the electrodes above and below the sample, which then leads to the motion of polarizable groups and an alternating current arises [31]. Then, the relevant parameters of the examined samples, such as permittivity, conductivity, and impedance, can be obtained owing to the movement of charged groups to overcome the stimulus of continuous changing fields [32,33]. Furthermore, these dielectric parameters characterize many mechanisms, for instance, interfacial polarization, phase balance, and relaxation behavior [34].

When it comes to BDS studies of emulsions and other colloidal systems, Sjöblom et al. and Sen et al. reported that both the permittivity at low and high frequency and relaxation time could be used to obtain information on flocculation in emulsions caused by electric fields [35,36]. Moreover, permittivity in BDS can monitor the phase separation more effectively compared with conductivity measurements [30]. The BDS study of the emulsion can be equivalent to the form of a resistor with a capacitor in parallel (Boyle model) [37], while phase separation can be described by a serial capacitor analogy. As emulsifiers accumulate at the interface, the conductivity of the emulsifier is more prominent than that of the interface, irrespective of the capacitances of the interface barrier [38]. However, emulsions are only metastable because the lowest energy state would have to be exactly one aqueous and one non-aqueous phase. Thus sedimentation, flocculation, and coalescence have a profound influence on droplet stability, which can be countered to some degree by surface engineering (surfactants, Pickering emulsifiers, …) [30,39].

There is a broad application for nanocellulose emulsions prepared by using CNC [12], MFC [40,41,42], BNC [43,44], and CNF [45]. Recently, the viscosity enhancement in emulsions upon introduction of nanocellulose as Pickering emulsifiers has been of great interest. These nanocellulose emulsifiers reside at the oil–water (O/W) interface and are interesting as they are renewable and nontoxic emulsifiers [46,47]. CNF can stabilize emulsions by forming a shell around the droplets, creating a steric barrier, and stabilized emulsions exhibit rigid solid-like viscoelastic properties under low shear forces, even at low CNF contents [19]. This is due to the formation of a highly entangled network, and its viscosity increases with a higher CNF content, which leads to a remarkable shear-thinning behavior [18,20]. Dickinson et al. [48] proved that those hydrocolloid-coated droplets could be immobilized by the surrounding polymer network and cause gelation, showing the yield behavior if the polymer content increases; in contrast, the whole system could become unstable because of weak connection throughout the droplets at low polymer contents. However, an excess of stabilizer cannot further promote the emulsion stability, arising from the possibility of dominating interparticle adsorption or flexible chains’ entanglements [49,50].

The viscosity studies of CNF emulsion or dispersions with different auxiliary ionic surfactants or CNF content had similar results as the work of Li et al. [18], which is corroborated by optical and confocal laser microscopic and dielectric research for this paper.

This paper investigates cellulose nanofiber Pickering emulsions with state-of-the-art physico-chemical characterization methods to obtain an insight into their behavior by broadband dielectric spectroscopy and rheology. These will be compared to different visualization techniques to assess the morphology and component distribution. This article is part I of this article series, which focuses on the characterization with rotational rheology, while part II will focus much more in-depth on the rheological properties, especially in dynamic mechanical test modes.

## 2. Materials and Methods

### 2.1. Materials

Cellulose nanofibers with a diameter of 5–100 nm, crystallinity of 70–90%, and length of >1 μm, dispersed in water at a concentration of 4.5 wt.%, were obtained from Zhongshan Nano bio-materials Co., Ltd. (Zhongshan, China). The raw CNF dispersion was diluted to the desired concentration. These dilutions were used for all aqueous-phase CNF emulsions. Didodecyldimethylammonium bromide (DDAB, Aladdin, Shanghai, China) dissolved in chloroform (Aladdin, Shanghai, China) was used as the secondary emulsifier to enhance the stability of CNF at the O/W interface. The oil phase was prepared by mixing 9 parts (by volume) liquid paraffin (Macklin, Shanghai, China) and 1 part chloroform DDAB solution. The compositions of the analyzed CNF emulsion samples are shown in Table 1. The mixed oil phase was added to the aqueous phase until an oil-to-water ratio of 1:2 was achieved. Then, the separated phases were emulsified by mechanical homogenization at 11,600 rpm for 3 min using a high-speed homogenizer (IKA T-10, Frankfurt, Germany). Meanwhile, the CNF aqueous dispersion samples with 0, 0.1, 0.3, and 0.5 wt.% DDAB content were prepared for steady rheological analysis. The rheological analysis was executed analogously to the startup-flow test of the emulsion samples and was used to prove the degree of CNF entanglement in the aqueous phase. The appearance of all samples with varying CNF content are shown in Figures S1 and S2, which show the yield stress (the samples do not sag under their own weight) except for the CNF0.5 and 1%-DDAB0.1% samples. The degree of dilution with water can be calculated by Equation SI1. The determined values are listed in Table S1.

### 2.2. Methods

**Microscopy.** Polarized optical microscopy (POM) images of CNF emulsions were investigated by an Axio Scope A1 polarized optical microscope (ZEISS, Scope A1, Oberkochen, Germany). Distinct and sparse CNF structures were captured by preserving a diluted CNF solution (ca. 0.8 wt.%) at a negative stain, which can be visualized using transmission electron microscopy (TEM) images (JEOL-2100F, Tokyo, Japan) to evaluate their nanofibers’ network and conformational states. Due to the specific fluorescence property of liquid paraffin, the droplets’ shapes can be easily observed. These droplet shapes were analyzed using fluorescence microscopy (Nikon, ECLIPSE Ti2-E, Tokyo, Japan) at an excitation wavelength of 355 nm and the DAPI channel at a wavelength of 460 nm for the emission wavelength. To gain several clear images of CNF distribution in the water phase, CNF chains were stained with a small amount of Calcofluor white, followed by detection with confocal laser scanning microscopy (ZEISS-LSM880, Oberkochen, Germany) in order to study the polymer distribution in the aqueous phase at different DDAB contents. Scanning electron microscopy images (Hitachi SU-70, Tokyo, Japan) of different dried (i.e., water-free) CNF emulsions visualize the structures. Unlike for the other emulsions in this paper, these images were taken using room-temperature solid paraffin, which otherwise is identical in terms of interfacial properties to avoid changes to the morphology. The solid samples were prepared by emulsifying at 80 °C at 11,600 rpm for 3 min using a high-speed homogenizer (IKA T-10, Frankfurt, Germany). After that, the heated emulsion cooled down and became completely solid, then the remaining water was removed by freeze-drying.

**Dielectric spectroscopy.** The CNF emulsions were measured using BDS (Concept 40, Novocontrol Technologies, Montabaur, Germany). The samples were between two gilded copper electrodes (diameter 20 mm, gap 0.1 mm, which was set by quartz rods with defined diameter, embedded in the sample), as shown in Figure S3. In a dielectric or impedance measurement, a voltage of 1.0 *V_rms_* (i.e., root-mean-square voltage) with a frequency (f) is applied to a sample cell. The electrical properties of CNF emulsion are measured at constant room temperature (T = 25 °C) and frequency sweeps, with an AC electric field of 1.0 *V_rms_* and a variable frequency f = 10^−2^…10^7^ Hz, starting at the highest frequency. As the emulsions are deformed during sample loading, it takes a while to reach an equilibrium state. For this reason, the abovementioned frequency sweeps are repeated until the equilibrium state has been reached, usually after 1–2 h. In general, 5 frequency sweep cycles (lasting ca. 12 min), corresponding to ca. 1h, were sufficient to reach an equilibrium response (Figure S4). Only the data in equilibrium were further evaluated.

**Rheology.** Rheological experiments were conducted on an Anton Paar MCR 302 (Graz, Austria) equipped with a Peltier heated lower plate and a Peltier hood. This heating system was tightly closed and the air in the oven was saturated with water to minimize sample evaporation. Measurements were carried out at 25 °C using a 50 mm/1° cone and plate geometry (CP50), with a gap (cone truncation) of 102 µm, avoiding bridging successfully. The rheological protocol is startup flow, as the basic flow behavior includes two up and down loops of shear rate ramps ($\dot{\gamma }$ = 0.01…1000 s^−1^/1000…0.01 s^−1^ with a logarithmic increase or decrease of $\dot{\gamma }$ and a ramp time of 920 s, Table S2). While the differences between runs 2, 3, and 4 are usually relatively small, only the data of the last run will be discussed. The resulting viscosity functions *η*($\dot{\gamma }$) and shear stress functions *τ*($\dot{\gamma }$) as well as the normal force functions *F_n_*($\dot{\gamma }$) were evaluated with respect to obtaining the yield stress $\tau_{y}$, the viscosity/stress at a high shear rate, and the normal force differences F_n_($\dot{\gamma }$ = 1000 s^−1^) vs. F_n_($\dot{\gamma }$ = 0.01 s^−1^).

These results were compared with the startup experiments of the multiple interval thixotropic test (miTT), which was described in detail by us before [51]. Essentially, the data were obtained by shearing the sample at constant $\dot{\gamma }$ for 120 s and taking the last datapoint as viscosity. Between each test, a dynamic mechanical test in the linear viscoelastic regime was performed, which is not evaluated further for this paper but which will be discussed in detail in part II of this article. To ensure that the data are comparable, the shear rates were tested in decreasing order.

**Zeta potential.** CNFs dispersed in DI water have a Zeta potential (Malvern Zetasizer Nano ZSP, Malvern, United Kingdom) of ca. −10.9 mV. By addition of even minute amounts of DDAB, the Zeta potentials are altered to around +9–11 mV (Figure S8) due to adsorption of the positively charged surfactant molecules. The standard deviation of 3 samples was around ±1 mV.

## 3. Results

### 3.1. Morphological Observations

**Figure 1:**
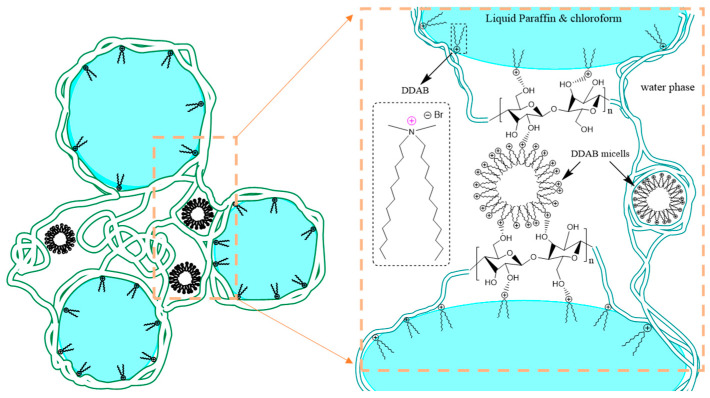
Schematic representations of CNF emulsion droplets, including the three CNF-crosslinked droplets, adsorbed DDAB molecules, and attached micelles, and the corresponding detailed interfacial structure showing hydrogen bonding between the DDAB molecules and the CNF.

The interfacial structure of the stable emulsion is shown in Figure 1. It comprises CNF chains and adsorbed surfactant molecules of DDAB, which are located at the oil–water interface, stabilizing the oil (liquid paraffin–chloroform mixture) in the water emulsion. The proposed stabilization mechanism is shown in Figure 1. The positive charge of the DDAB is conducive for the stabilization of the Pickering emulsion with the CNF, as electrostatic forces between the auxiliary stabilizer and the CNF further stabilize the interfaces. Moreover, DDAB can be present in the system as regular micelles (in the aqueous phase) or as inverse micelles in the oil phase. CNF and DDAB interact with each other through the charged DDAB head group (N^+^) and the O^2−^ of CNF. (Please note: the single green chain drawn in Figure 1 represents thin membranes forming by numerous long-scale units of CNF molecules, which assemble a dense coating layer at the interface.)

#### 3.1.1. Polarized Optical Microscopy

**Figure 2:**
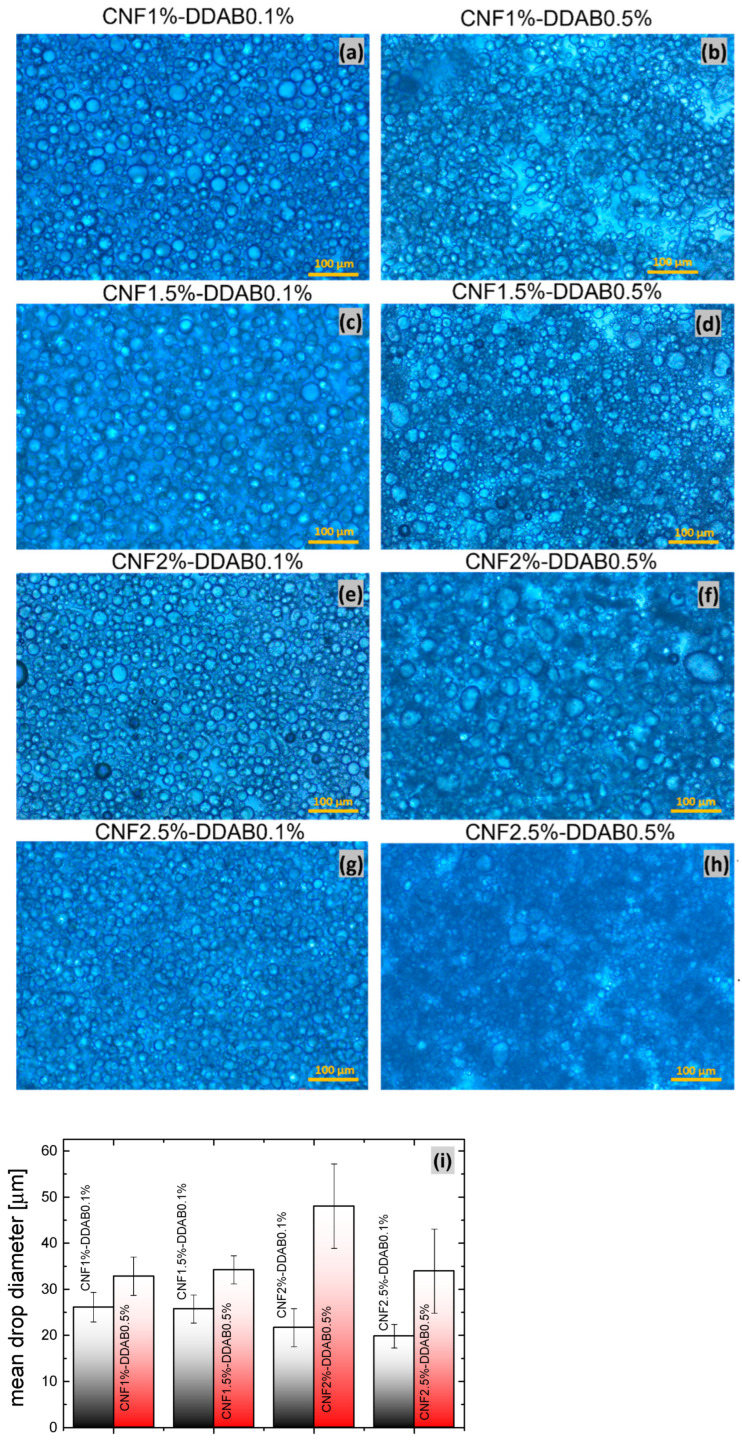
Optical microscopy characterization of (**a**,**b**) CNF1%-DDAB0.1 and 0.5%, (**c**,**d**) CNF1.5%-DDAB0.1 and 0.5%, (**e**,**f**) CNF2%-DDAB0.1 and 0.5%, and (**g**,**h**) CNF2.5%-DDAB0.1 and 0.5%. Contrast adjusted for improved visibility. (**i**) Column graph concerning the droplets’ mean size. Sizes were determined by Image J.

Figure 2a–h show the optical microscopy images of several CNF emulsions with CNF content of 1%, 1.5%, 2%, and 2.5% and DDAB content of either 0.1% or 0.5%. The droplets are generally round and exhibit a homogeneous size with diameters between 20–50 µm, demonstrating the high stability of the interfacial films brought about by the CNF. For the sample series with 0.1% DDAB content, the droplet size is more homogenous and is smaller with increasing CNF content (Figure 2j), which was related to the limited coalescence regime affecting the formation of smaller droplets at higher CNF contents [45,52]. For the 0.5% DDAB content series, however, the droplet size is larger and relatively constant but lacks size homogeneity, which becomes obvious from the larger error bars. The error bars are caused by some droplets in the 0.5% DDAB content series being significantly larger than the others. Furthermore, they are also less spherical due to their large size and decreased surface tension. Moreover, a regional giant dark shadow can be noticed in all DDAB0.5% samples, which is indicative for dense accumulation of flexible CNF chains distributed in the aqueous phase. However, the limitation of polarized optical microscopy (POM) images is that it is not possible to visualize CNF agglomeration or segregation in the aqueous phase further. Figure 2f–h shows the multi-scale droplet distribution, containing plentiful droplet sizes smaller than 10 μm and larger than 50 μm. In presence of DDAB, the strong association between CNFs and surface will obviously increase the interfacial modulus and probably form a larger interfacial area [28]. For the droplets smaller than 10 μm, it is possible for the DDAB micelles to form a shell around the larger droplets stabilized by CNF chains, resulting in the mixture of low and high levels of droplets, then contributing to coalescence stability [53].

#### 3.1.2. Fluorescence Analysis on CNF Distribution

**Figure 3:**
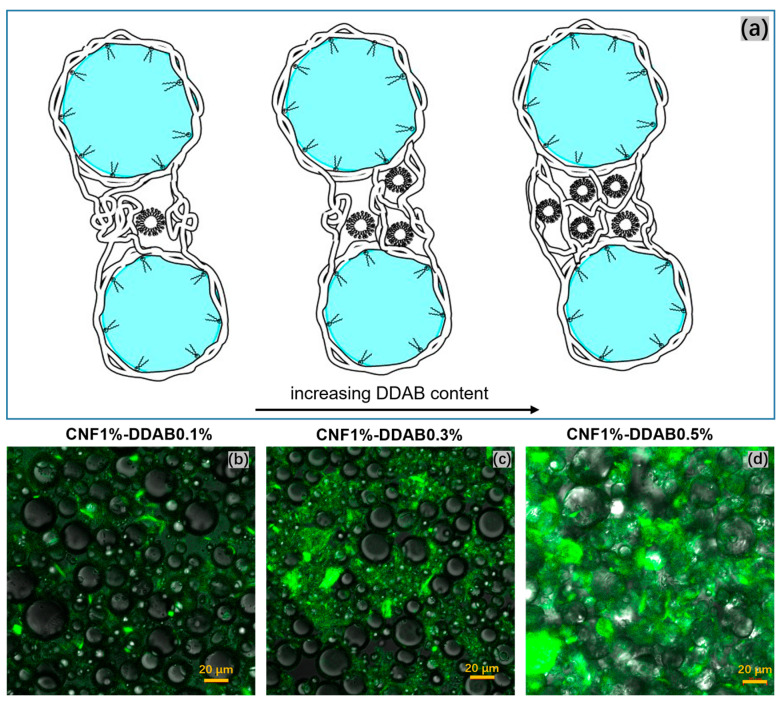
(**a**) Schematic representations of CNF distribution in aqueous phase with increasing DDAB micelle number (increasing DDAB concentration). Confocal laser scanning microscopy images of CNF emulsion with 1 wt.% CNF and (**b**) 0.1, (**c**) 0.3, and (**d**) 0.5 (wt.%) DDAB after being stained with Calcofluor white, which shows the green areas of spreading CNF chains over aqueous phase.

The morphology of the dense CNF aggregates was investigated by confocal laser scanning microscopy with an excitation wavelength of 355 nm and an emission wavelength of 430 nm. For the similar research based on this imaging technology, Jiang et al. [54] have reported that isolated drops stabilized by regenerated cellulose (RC) and carboxylmethyl cellulose (CMC) were labeled with Calcofluor White and they succeeded in observing the isolated drops with fluorescent surfaces. Figure 3b,c show the green fluorescence emission of the CNF, which is distributed in the aqueous phase. In contrast, the spherical oil droplets do not show fluorescence because paraffin and DDAB do not fluoresce at this wavelength [54,55], thus, the distinct cluster areas are directly seen [49]. When the DDAB content increases, the fluorescent region, which represents the CNF distribution, expands merely in the aqueous phase. At high DDAB concentration, the CNF partially obscures the droplets’ view, which directly indicates that CNF emulsions become thicker and harder to flow. Consequently, CNF chains in the aqueous phase can bridge several oil droplets and form small micelles (without oil droplets inside), as shown in Figure 3a. However, when the DDAB content increases, the CNF chains tend to separate against entanglement by itself, resulting in a stronger charged connection using DDAB micelles and smaller CNF flocs. These networks are displayed in the third schematic illustration in Figure 3a and contribute to higher viscosity and harder flowability, which affect the yield stress and would be proved in rheological studies.

#### 3.1.3. SEM Analysis on CNF Droplets

**Figure 4:**
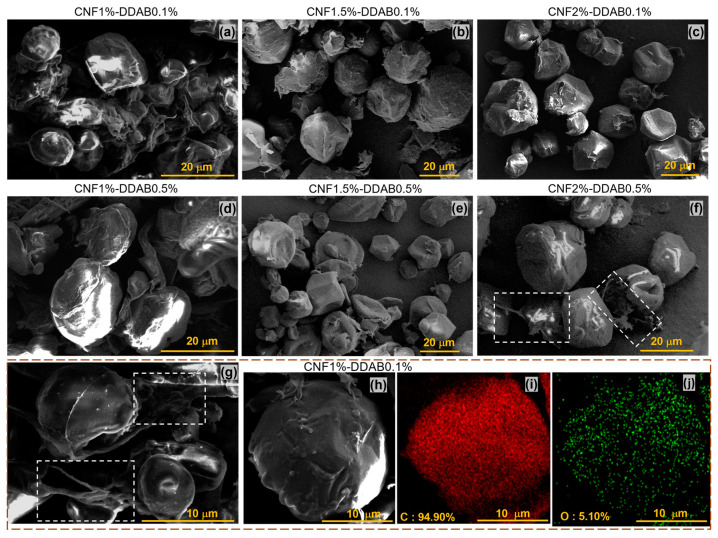
SEM images of CNF emulsion droplets for CNF contents in (**a**) 1%, (**b**) 1.5%, and (**c**) 2% at 0.1 wt.% DDAB content and in (**d**) 1%, (**e**) 1.5%, and (**f**) 2% at 0.5 wt.% DDAB content. (**g**) Another SEM image of CNF1%-DDAB0.1% shows obviously the aqueous CNF chain as the bridge connecting between droplets. Its EDS maps showing the elemental content and distribution of (**i**) carbon and (**j**) oxygen for the individual droplet are shown in (**h**). The white dashed wireframe in (**f**,**g**) mark the flexible polymer chains outside the solid droplets.

SEM images of six dried CNF emulsions with three different CNF contents and two different DDAB contents are shown in Figure 4a–f. The roundest particles are found for CNF1.5%-DDAB0.1% (Figure 4b), indicating a stable interface, while CNF1%-DDAB0.1% and CNF1%-DDAB0.5% appear to have a less stable interface as partially disintegrated droplets can be seen (Figure 4a,d). Higher CNF and DDAB contents lead to inward-dented surfaces of the droplets, which resemble soccer balls with too little pressure (Figure 4c,e,f). This can be explained by the fact that DDAB was introduced into the oil phase in a chloroform solution; thus, up to 10% of the oil phases’ volume is lost during the drying process, which leads to these deformed spheres if the interface is strong enough to resist shrinking. Indeed, the solid droplets’ appearance after vacuum freeze-drying is affected not just by cooling degrees but also by the drying temperature and time, which influence the dynamic CNF interfacial self-assembly in response to water evaporation as well as chloroform evaporation from the paraffin phase.

When focusing on the chains on the droplets’ surfaces of CNF2%-DDAB0.5%, the CNF networks can be clearly seen as well as bridging to neighboring droplets (Figure 4f), which is also observed at CNF1%-DDAB1% (white dashed wireframe, Figure 4f,g). Corresponding to Figure 3b–d and Figure 4f,g, some CNF chains can maintain their appearance after drying and exist on the “ball” surface. Figure 4i shows these features for CNF1%-DDAB0.1% along with the energy dispersive X-ray spectroscopy (EDS) maps (Figure 4i (C), Figure 4j (O)), showing a homogeneous distribution of both atoms on the droplet. While paraffin, DDAB, and CNF obviously contain carbon, oxygen is only a minor fraction in paraffin and DDAB (theoretically 0). However, CNF contains carbon and oxygen in a 6:5 atomic ratio (the repeat unit of cellulose is C_12_H_20_O_10_). Further, traces of water are possibly present in the sample as well. Thus, the determined amount of 5.1 wt.% oxygen (Figure 4j) suggests coverage of the droplet with CNF but does not prove it because of the aforementioned alternative explanations. However, the theoretical oxygen content of CNF is much higher, so, in EDS mapping, a combination of the CNF, DDAB, and paraffin signals is observed.

The emulsion of CNF1%-DDAB0.1% has smaller particle sizes owing to the rather low CNF content being unable to stabilize larger droplets. The obvious shrinkage of the CNF shell around the droplets due to drying (and thus the evaporation of the chloroform) leads to a relatively flat and collapsed structure, unlike, e.g., CNF2%-DDAB0.1%, suggesting that the droplets’ coverage with CNF is relatively thin, which, therefore, is not very stable against shrinkage of the droplet below the surface. For higher CNF contents, such as CNF2%-DDAB0.5%, the thicker CNF shell can not only stabilize larger droplets sufficiently but is also able to connect droplets.

### 3.2. Dielectric Spectroscopy

When an AC electric field stimulates the CNF emulsion, the charged and polarizable groups of CNF chains and DDAB will show a characteristic response, which allows for extracting information on the emulsion properties. The parameters $\sigma^{*}$, $\varepsilon^{*}$,$\;z^{*}$, $\delta_{min}$, and |φ| correspond to complex conductivity, permittivity, impedance, the lowest shift factor calculated from the real and imaginary part of permittivity, and phase angle of AC current and voltage, respectively. Their relevant functions are illustrated in the Supplementary Information of this work.

**Figure 5:**
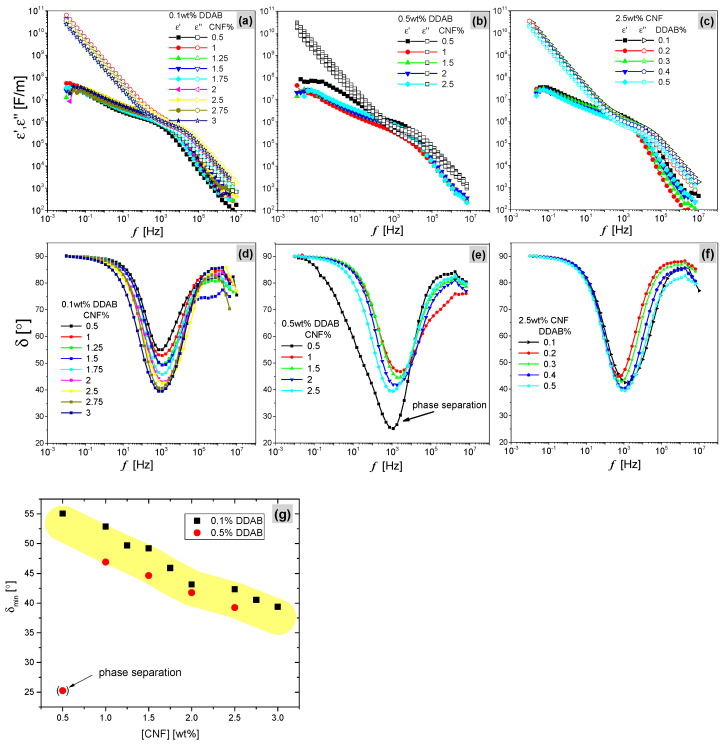
Graphs of permittivity and loss angle dependence on frequency for (**a**,**d**) CNF series with 0.1 wt.% DDAB, (**b**,**e**) CNF series with 0.5 wt.% DDAB and (**c**,**f**) DDAB series with 1 wt.% CNF, respectively. (**g**) $\delta_{min}$ as the function of CNF content and the phase-separated sample show an obvious unfitting.

Figure 5a–c show the plots of $\varepsilon^{\prime }$ and $\varepsilon^{\prime \prime }$ as a function of f after combining the data of the last loop for all CNF emulsions, which obey Debye’s classical equations (Equation (SI7)) [37,56]. All emulsions show similar characteristics with different CNF or DDAB contents and increasing $\varepsilon^{\prime }$ and $\varepsilon^{\prime \prime }$ in lower f, which indicates the strong polarization dependance on f owing to $\varepsilon^{\prime }\left(\omega \right)\sim P$ according to Equation (SI6) [57]. More importantly, the curves of $\varepsilon^{\prime }\left(f\right)$ and $\varepsilon^{\prime \prime }\left(f\right)$ are divided into two parts at $f=10^{3}$ Hz, where $f<10^{3}$ shows the deviation curves associated with lower slope of $\varepsilon^{\prime }$ but $f>10^{3}$ shows them closely overlapping, indicating the different relaxation domains [36]. In order to directly analyze the two domains, phase angle δ as a function of *f* for all emulsions is plotted in Figure 5d–f according to Equation (SI7) and Equation (SI8), which shows very high damping (δ close to 90°) in most of the frequency ranges, except for a minimum in the middle of the frequency range. The minimum δ(f) of all CNF emulsions is located around $f=10^{3}\sim 10^{4}$ Hz and both sample series (0.1 and 0.5 wt.% DDAB) display a decrease in the δ-minimum ($\delta_{min})$ with increasing CNF content. In contrast, emulsions stabilized with GO particles show a different behavior associated with $\delta_{min}$ (Figure S5). δ>45°, corresponding to $\varepsilon^{\prime }<\varepsilon^{\prime \prime }$, means that more energy is dissipated than stored. This explains the stronger heat loss during the dielectric test, because the testing samples are carbonized gradually (Figure S6). CNF emulsions with lower CNF content display a remarkable hysteresis, owing to the higher δ and the decreasing charge density [58]. The content of the oxygen-containing functional group mainly affects the electric properties of CNF emulsions [59]. Moreover, increasing the DDAB content at the interface can promote the adsorption of CNF on oil droplets, leading to a slightly decreasing loss angle (Figure 5g). Moreover, due to the obvious phase separation of CNF0.5%-DDAB0.5% before loading this sample, the BDS behavior can be visualized, and it can be concluded that the dielectric characteristics are a sensitive means to monitor phase changes.

The BDS results can be summarized as follows. All samples except for 0.5% DDAB 0.5% CNF showed very similar behavior patterns, but the characteristic minimum of δ and its position on the frequency axis varied systematically, which can be understood as the material systematically hindering the dissipation of the specific frequency, which is stronger when more CNF and DDAB are in the sample. 

The characteristic data of $\left|\sigma^{*}\right|$, |$\varepsilon^{*}|,$ and $\left|z^{*}\right|$ are listed in Table 2 based on the increasing trends of CNF or DDAB content. Clearly, $\delta_{min}$ decreases with increasing CNF, suggesting that the mobility decreases, especially in the aqueous phase, where most of the dipoles exist. The characteristic frequency of the minimum, however, is much less varied. When the DDAB content is increased, $\left|\sigma^{*}\right|$ and $\left|\varepsilon^{*}\right|$ show decreasing trends opposite to $\delta_{min}$ and |φ|, as expressed in Equations (S4) and (S5) [38,60]. However, the absolute values of complex impedance stay relatively constant except for the emulsion systems (CNF0.5%-DDAB0.1% and 0.5%).

The CNF and DDAB concentration-dependent dielectric properties of CNF emulsions were investigated at 25 °C in the frequency range of 10^−2^ to 10^7^ Hz. At $f=10^{3}\sim 10^{4}$ Hz, the minimum values corresponding to the polarization relaxation frequency strongly depend on increasing the CNF content but are slightly influenced by DDAB content. The complex permittivity of larger CNF contents increases $\delta_{min}$ because of the greater stimulation of ionic transfer in the AC field and polarization when introducing more CNF chains. The higher the DDAB content and the higher the CNF content, the higher the viscosity of the sample and thus the possibility to store polarization elastically, lowering $\delta_{min}$.

### 3.3. Rheology

#### 3.3.1. Deformation-Induced Morphology Changes

**Figure 6:**
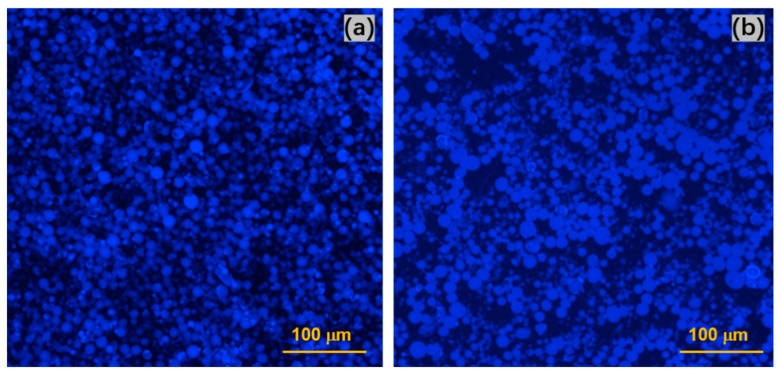
Fluorescence images of CNF2.5%-DDAB0.1% (**a**) before and (**b**) after deformation at a wavelength of 460 nm that was captured by the constant shear from 0.1 s^−1^ to 1000 s^−1^.

The arrangement of droplets before and after deformation can be investigated by fluorescence microscopy because of the paraffin’s strong fluorescence at a wavelength of 460 nm and ideal depth of field (Figure 6). Comparing the droplet sizes of the sample CNF2.5%-DDAB0.1% before deformation with the end of high shear rate, the droplets maintain the shape, scale, and size, however, the assembled features of bulk droplets tend to approach each other, resulting in the formation of a larger space of the aqueous phase (Figure 6a). Kalashnikova et al. [19] have proved that the longer the length of the CNC nanocrystals, the more aggregated droplets clusters are formed. In other words, entangled systems of interconnective droplets are created. Nomena et al. [61] illustrated that un-adsorbed soluble biopolymers can create a viscoelastic network in order to prevent droplets from creaming, and then contribute to attractive depletion interaction. Moreover, the stronger CNF networks contribute to the elastic deformation of the emulsion and their entanglement cannot be destroyed totally in the aqueous phase after exposition to high deformations [45,62]. Therefore, the residual recovery force is responsible for the formation of droplet clusters.

#### 3.3.2. Startup Flows

**Figure 7:**
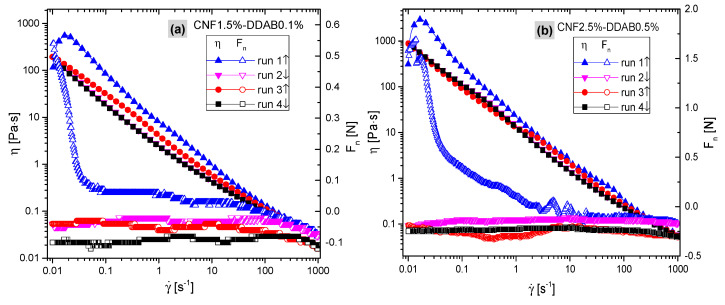
Viscosity (left axis) and normal force (right axis) as the function of shear rate at 1~4 ramps, showing the samples of (**a**) CNF1.5%-DDAB0.1% and (**b**) CNF2.5%-DDAB0.5%, respectively.

For multiple shear ramp profiles, four shear ramps (runs) were performed (run one: $\dot{\gamma }$ = 0.01 s^−1^→1000 s^−1^, run two: $\dot{\gamma }$ = 1000 s^−1^→0.01 s^−1^, run three: $\dot{\gamma }$ = 0.01 s^−1^→1000 s^−1^, run four: $\dot{\gamma }$ = 1000 s^−1^→0.01 s^−1^, no intermissions between them), which lasted 920 s each. None of the samples showed a clear normal force increase or decrease beyond ±0.3 N upon shearing (except for startup effects). The higher viscosity samples showed a slight decrease of normal force at $\dot{\gamma }>$ 100 s^−1^ (Figure 7). Please note that the normal force can only be measured with a precision of 0.01 N with the current setup—thus, its step-like appearance. The first (and sometimes the second) startup flow ramp is always significantly higher than the following and is almost identical in most cases (Figure 7). Thus, only the last segment—run four—was used for further evaluation.

The normal forces (open symbols) show a sharp decrease for $\dot{\gamma }$ below 0.1 s^−1^ in the first run, usually followed by a minor further decrease, which is due to the initial yield structure being broken up by the shear. More importantly, a decrease in the normal force was observed at $\dot{\gamma }>$ 100 s^−1^, which is atypical of shear-thinning materials, except those with a plate or rod-like nanoparticles in them. This suggests that the effect of droplet deformation, which also decreases the normal force at high $\dot{\gamma }$, is less important than the influence of the CNF. The changes in normal force by ca. −0.1 N correspond to a first normal stress difference *N*_1_ of ca. −51 Pa. For higher DDAB contents, the effect is significantly smaller but still visible.

**Figure 8:**
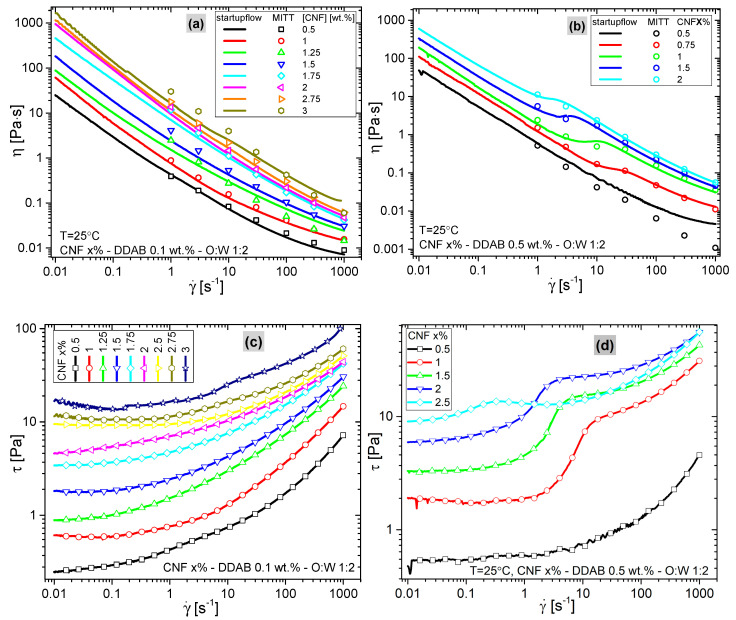
Startup flows in comparison with viscosity functions determined with the miTT protocol. Viscosity functions of (**a**) the 0.1 wt.% DDAB series and (**b**) the 0.5 wt.% DDAB series. Shear stress functions of (**c**) the 0.1 wt.% DDAB series and (**d**) the 0.5 wt.% DDAB series.

**Figure 9:**
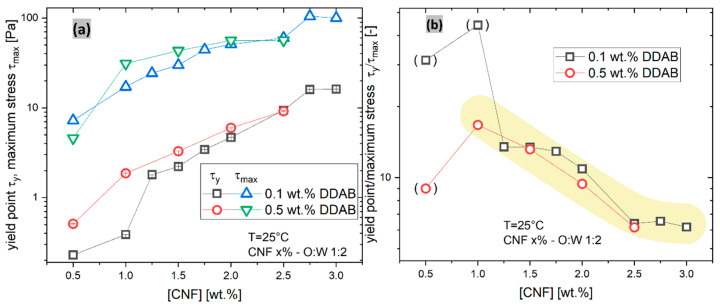
(**a**) Dependence of yield stress values $\tau_{y}$ on the CNF or DDAB composition. The error bars are the uncertainties of the fit. (**b**) Relation between viscosity at 10 s^−1^ and emulsion constituents.

Figure 8 shows the results of the startup flows, determined by the multiple shear ramp profiles and the startup flows of the miTT test. For the multiple shear ramps, only the fourth ramp ($\dot{\gamma }$ = 1000 s^−1^→0.01 s^−1^) is shown in all cases. The SI shows some examples, that is CNF3%-DDAB0.1% and CNF1%-DDAB0.5%, of the raw startup flow test within *τ* and *F_N_* as the function of $\dot{\gamma }$ of the 1–4 runs (Figure S7). The viscosity functions *η(*$\dot{\gamma }$*)* of the samples with 0.1 wt.% DDAB (Figure 8a) appear relatively standard for a material with a yield point. Clearly, adding more cellulose (CNF) increases the viscosity, but at first glance, the shape of the viscosity functions is not significantly influenced by the changes in composition. A comparison between the multiple shear ramps and the miTT startup flows shows a good agreement (±10%), demonstrating that both protocols deliver reliable values. When plotting the same data in terms of shear stress functions *τ(*$\dot{\gamma }$*)* (Figure 8c), it becomes obvious that, for the lowest shear rates $\dot{\gamma }$, *τ* approximately approaches a constant value, which can be understood as the yield stress $\tau_{y}$, which increases with CNF content (cf. Figure 9). However, when looking at *τ*($\dot{\gamma }$) for low shear rates in detail, it is obvious that the higher the CNF content, the higher the shear rate $\dot{\gamma }$, at which a more or less constant value is reached and for the highest CNF contents even a small increase towards the smallest $\dot{\gamma }$ is found, which indicates a structural recovery from the high shear rates imposed on the sample before.

When comparing the data of the samples with 0.5 wt.% DDAB (Figure 8b,d) with those with 0.1 wt.% DDAB (Figure 8a,c), the samples with 0.5 wt.% DDAB (except for the CNF0.5%-DDAB0.5%-O:W 1:2 sample, which is very similar to CNF0.5%-DDAB0.1%-O:W 1:2) show a clear kink between $\dot{\gamma }$ = 10 and 0.2 s^−1^, which is visible for miTT data as well. This clear difference suggests that the 0.5 wt.% DDAB has some structural changes that cause this unusual curve shape. This kink is even more visible as a step in *τ*($\dot{\gamma }$) (Figure 8d) and it is found for all four ramps in the experimental setup (Figure S5). For the same experiments on similar samples, Li et al. [18] and Jia et al. [63] found analog steady-state viscosity curves and divided them into four regions: fibers’ orientation, fibers’ entanglement, networks’ breakdown, and well-oriented structure.

Considering this difference in rheological data when increasing the surfactant content fivefold (0.5 wt.% DDAB) and that it only happens when the CNF content is sufficiently high (>0.5 wt.%), it is clear that the viscosity increase has to be related to the stability of the interfaces, as those are strengthened by increasing surfactant (DDAB) and Pickering emulsifier/surfactant (CNF) contents. Interestingly, the highest step is found for 1% (Figure 8d), while higher CNF contents decrease the step significantly. The step could be interpreted as an increased viscosity due to a high-shear-rate structure that collapses to a low resistance configuration once the shear rate $\dot{\gamma }$ is below this critical shear rate at the step (for CNF 1 wt.%, it is in the shear rate range of 3–20 s^−1^).

In order to better compare the multitude of data, two characteristic quantities were defined:

1. The maximum stress $\tau_{max}$ was directly determined as the maximum stress recorded (always found at a shear rate of $\dot{\gamma }$ = 1000 s^−1^). Higher shear rates destroyed the sample and were therefore not included in the paper.
2. The yield stress $\tau_{y}$ was determined using a yield-stress modified Carreau–Yasuda model [64]
  (1) $$\eta \left(\dot{\gamma }\right)=\eta_{0}{\left(1+{\left(\dot{\gamma }/{\dot{\gamma }}_{c}\right)}^{a}\right)}^{\frac{n-1}{a}}+\tau_{y}/\dot{\gamma ,}$$
  where ${\dot{\gamma }}_{c}$ denotes the characteristic shear rate, a denotes the sharpness of the transition in a regular viscosity function, n denotes the slope of the viscosity function at a high shear rate (n = 0→dlog*η*/dlog$\dot{\gamma }$ = −1), and $\tau_{y}$ denotes the yield stress. As the contribution of $\tau_{y}$ dominates the viscosity function at low $\dot{\gamma }$, the other parameters were not evaluated, owing to their high dependence on each other. For the 0.5% DDAB sample series with the kink in the data, the fitted yield stress values $\tau_{y}$ were checked with extreme care and found within the range that one would determine manually from visually extrapolating the data towards $\dot{\gamma }$ = 0.

Figure 9a compares the yield stress $\tau_{y}$ and the maximum stress observed $\tau_{max}$ (at $\dot{\gamma }$ = 1000 s^−1^). As already visible from Figure 8c,d, an increase in the CNF content leads to an increase in $\tau_{y}$. Clearly, the differences between the 0.1 wt.% and the 0.5 wt.% DDAB are diminishingly small at high CNF contents, while they are more significant at low CNF contents. The maximum stress observed $\tau_{max}$, which is always observed at the highest shear rate of $\dot{\gamma }$ = 1000 s^−1^, shows a relation which appears to be similar with $\tau_{y}$ at a first glance, about 10 times the $\tau_{y}$ value. However, when plotting the ratio of $\tau_{max}/\tau_{y}$ vs. CNF content (Figure 9b), it becomes clear that the higher the CNF content, the lower $\tau_{max}/\tau_{y}$ is, and it does not depend significantly on DDAB content. For 0.5 wt.% CNF, 1 wt.% CNF, and 0.1 wt.% DDAB (points in bracket), the relation does not show reliable values, which we attribute to the low yield stress $\tau_{y}$, making a very precise determination difficult.

These findings can be explained by both CNF and DDAB stabilizing the interfaces. The emulsion consists of a water matrix with oil droplets and the interfaces by DDAB and CNF. The yield stress $\tau_{y}$ is the resistance of the material against any flow, i.e., below $\tau_{y}$ the sample behaves as a solid. The yield point $\tau_{y}$ depends on how many of the droplets are interlocked, i.e., how stiff their interfaces are. Very soft interfaces make it very easy for the droplets to slide past each other, while infinitely hard interfaces could theoretically lead to jamming [16,18]. DDAB as a normal surfactant stabilizes the interface as a classical amphiphile, i.e., by sitting at the interface with the hydrophilic head in the aqueous phase and two hydrophobic tails in the oil phase. As a general rule, increasing the amount of amphiphile decreases the droplet size as coalescence is reduced due to the improved stabilization of the interface [19,44,65].

The relations in Figure 9 can be interpreted as the consequence of CNF stiffening the droplet surface, thus increasing the $\tau_{y}$ in an approximately exponential fashion, making the yield stress well-tailorable. The stress at $\dot{\gamma }$ = 1000 s^−1^ shows a similar relation but with a lower slope, so that the ratio $\tau_{max}/\tau_{y}$ decreases with increasing CNF content. This can be interpreted with the yield stress $\tau_{y}$ being more strongly influenced by the stiffening of the interface than the behavior under strong shear. This can be explained by the structures hindering the flow being shear sensitive, i.e., they lose their strength upon applying a significant shear rate. Furthermore, the decreasing ratio $\tau_{max}/\tau_{y}$ means that, at high CNF contents, jamming does not play a significant role as that would lead to an increasing $\tau_{max}/\tau_{y}$ ratio.

**Figure 10:**
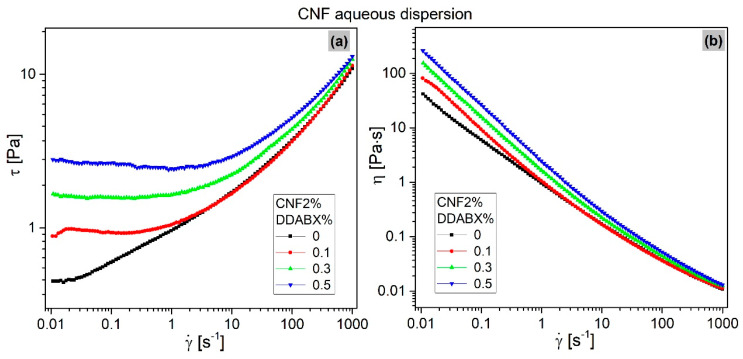
(**a**) Stress function and (**b**) viscosity function of CNF water dispersion with 2 wt.% CNF series.

However, the situation needs to be considered from the point of view of the influence of CNF suspensions, too. For this purpose, 2 wt.% CNF suspensions (without an oil phase) were made with DDAB contents of 0, 0.1, 0.3, and 0.5 wt.%, which will be discussed in the following sections. Those showed viscosity functions very similar to those shown in Figure 8a,b (see Figure 10). The yield stresses $\tau_{y}$ were found to be 1 Pa for 0.1% DDAB and 3 Pa for 0.5% DDAB, which are lower than the $\tau_{y}$ values of 5 Pa and 6.5 Pa found for the CNF2%-0.1%DDAB-O:W 1:2 and CNF2%-0.5%DDAB-O:W 1:2 emulsions, respectively. When considering that the CNF is mostly located in the aqueous phase of the emulsion, a comparison with the CNF1.25%-0.1%DDAB-O:W 1:2, CNF1.5%-0.1%DDAB-O:W 1:2, and CNF1.5%-0.5%DDAB-O:W 1:2 emulsions—having approximately the same concentration in the aqueous phase—should be conducted, too. Those samples yield stresses $\tau_{y}$ are found to be between 2 and 3 Pa, almost identical to the values found for the suspension. Thus, it is safe to conclude that the CNF offers a major contribution to the yielding stress in suspension and the emulsion’s oil–water interface-related processes.

In order to understand these results better, the non-emulsion equivalents of the emulsions were also tested in CNF dispersions with variable DDAB contents, i.e., these samples differ from all other samples in this paper by not having an oil phase. Figure 10a,b show the viscosity function of a 2 wt.% CNF dispersion with 0, 0.1, 0.3, and 0.5 wt.% DDAB content. It is clearly visible that the data at high $\dot{\gamma }$ are almost undistinguishable, while at low $\dot{\gamma }$, a high DDAB content leads to a quite significant yield stress $\tau_{y}$ of ca. 3 Pa (vs. ca. 6 Pa for CNF2% 0.5%DDAB O:W 1:2). At 0.1 wt.% DDAB content, only $\tau_{y}$ of ca. 1 Pa is found (vs. ca. 4.7 Pa for CNF2% 0.5%DDAB O:W 1:2). In comparison to the data for the Pickering emulsions, it is clear, therefore, that the yield stress $\tau_{y}$ varies significantly more and is lower. One might argue that CNF content in the aqueous phase of the emulsion is higher than 2% and, therefore, we have to compare the obtained yield stresses with those of emulsions with a lower CNF content (e.g., based on O:W 1:2), and an equivalent CNF content should be reached around 1.3 wt. % CNF content in the Pickering emulsions. However, such comparisons are difficult as the amount of the CNF and DDAB not in the aqueous phase in the Pickering emulsions cannot be precisely accounted for. It is clear, however, that the dependence of the yield stress $\tau_{y}$ in Figure 8c is much larger for the CNF dispersions (Figure 10a) than for the Pickering emulsions. This suggests an equivalent relation as plotted in Figure 8b cannot be set up, as no approximate DDAB content independent $\tau_{max}/\tau_{y}$ ratio exists, as can be also seen by comparing Figure 7 and Figure 9. Therefore, it is clear that the yield stress $\tau_{y}$ found for the Pickering emulsions is partially caused by the oil droplets and partially caused by the CNF–DDAB agglomerates in the aqueous phase. Those CNF–DDAB agglomerates are relatively weakly bonded, as can be seen from the fact that, at high $\dot{\gamma }$, the viscosities are almost identical.

## 4. Conclusions

A study of the properties of a Pickering emulsion with cellulose nanofibers (CNF) has shown that the behavior is determined by a combination of the properties of the regular surfactant DDAB in stabilizing the interfaces and of CNF stabilizing the interfaces as well as producing a colloidal network. The controlled variation of the DDAB content and CNF content has helped in unraveling the interdependencies between these different components, which are very complex due to their interrelations.

The behavior of the Pickering emulsions is clearly determined by CNF and DDAB stabilizing the oil–water interface; the presence of oxygen, proven by EDS, stems from CNF on the paraffin droplets, while the presence of DDAB can be indirectly derived from the changing morphology with increasing DDAB content (and the change in BDS and rheological properties, too). The two emulsifiers solidify the whole emulsion sufficiently to call it an emulsion. However, CNFs are not only localized at the oil/water interface but are also present in the form of more or less precipitated bridges and clusters in the aqueous phase. There, the CNFs are stabilized by DDAB (micelles). The rheological properties show that most of the material properties can be explained by combining these two contributions. Microscopy has shown that the structure is mostly stable, although intensive shear has led to some minor modifications. Broadband dielectric spectroscopy has shown that the higher the DDAB and CNF content, the more the samples can store energy (from dielectric polarization) elastically, which suggests that the interfaces become more organized but also that the aqueous phase, containing the largest number of polarizable groups (stemming from the water dipoles), is significantly affected in terms of its flowability (increasing viscosity). The best explanation for this is that CNF and DDAB molecules lead to the partial gelation of the aqueous phase. This could also be deduced from the fact that, for CNF suspensions, the viscosity increases with increasing DDAB content at the highest shear rates. Furthermore, this can also be related to the oil–water interface becoming stiffer and more crowded, thus leading to a better elastic storage as well.

This paper has given a first overview of the properties of CNF Pickering emulsions. Part II will cover a much more in-depth rheological characterization that will lead to even deeper insights with respect to the understanding of the detailed physico-chemical interactions. Together, these two papers will provide reliable information on how Pickering emulsions based on CNF can be designed to fulfil a certain application profile.

These emulsions can serve as model material for, e.g., 3D printing inks or also for the optimization of food in industrial processes.

## Figures and Tables

**Table 1:** Composition of analyzed CNF emulsions.

Sample	CNF (wt.%)	DDAB (wt.%)	O:W Ratio
CNF 0.5%-DDAB 0.1%	0.5	0.1	1:2
CNF 1%-DDAB 0.1%	1		
CNF 1.25%-DDAB 0.1%	1.25		
CNF 1.5%-DDAB 0.1%	1.5		
CNF 1.75%-DDAB 0.1%	1.75		
CNF 2%-DDAB 0.1%	2		
CNF 2.5%-DDAB 0.1% *	2.5		
CNF 2.75%-DDAB 0.1%	2.75		
CNF 3%-DDAB 0.1%	3		
CNF 0.5%-DDAB 0.5%	0.5	0.5	1:2
CNF 1%-DDAB 0.5%	1		
CNF 1.5%-DDAB 0.5%	1.5		
CNF 2%-DDAB 0.5%	2		
CNF 2.5%-DDAB 0.5% ^§^	2.5		
CNF 3%-DDAB 0.5%	3		
CNF 2.5%-DDAB 0.1% *	2.5	0.1	1:2
CNF 2.5%-DDAB 0.2%		0.2	
CNF 2.5%-DDAB 0.3%		0.3	
CNF 2.5%-DDAB 0.4%		0.4	
CNF 2.5%-DDAB 0.5% ^§^		0.5	

*^,§^ these samples are identical but listed twice for the sake of reading convenience.

**Table 2:** Values of minimum loss angle during the whole frequency range and corresponding |φ|, $\left|\sigma^{*}\right|$, |$\varepsilon^{*}|$, and $\left|z^{*}\right|$ dependence on different CNF or DDAB contents at same state.

CNF (wt.%)	DDAB (wt.%)	$\delta_{min}$ (°)	f (Hz)	| φ| (°)	|ε*|·10^6^ (F/m)	|σ*|·10^−3^ (S/m)	|z*| (Ohm)
0.5	0.1	55.01	916.6	34.99	1.29	0.54	7.21
1		52.87	1175.92	37.13	1.99	1.28	2.05
1.25		49.39	1188.99	40.61	1.20	0.77	3.32
1.5		49.27	1379.02	40.72	1.29	0.82	3.85
1.75		45.76	1203.48	44.24	1.08	1.07	2.21
2		42.91	1430.01	47.09	1.36	0.87	3.65
2.5		42.47	1412.81	47.53	2.16	0.90	3.54
2.75		40.66	1063.07	49.39	1.80	0.75	4.23
3		39.46	1099.05	50.54	1.94	0.81	3.93
0.5	0.5	25.44	1078.02	64.56	1.10	0.70	6.28
1		46.74	2596.59	43.26	0.32	0.49	5.30
1.5		44.59	2204.97	45.41	0.71	0.70	2.96
2		41.77	1552.12	48.23	0.72	0.71	3.94
2.5		39.40	994.74	50.60	0.96	0.62	3.97
2.5	0.1	42.47	1175.92	47.53	2.16	0.90	2.29
	0.2	44.82	494.67	45.18	2.39	0.65	4.91
	0.3	42.72	764.70	47.28	2.18	0.91	3.65
	0.4	40.28	770.09	49.72	1.72	0.72	2.71
	0.5	39.40	1159.51	50.60	0.96	0.62	3.38

## Data Availability

The data presented in this study can be obtained upon request by email from the authors.

## Supplementary Materials

The following supporting information can be downloaded at: https://www.mdpi.com/article/10.3390/ma15238285/s1, Table S1. The component ratio of aqueous phase including CNF gel weight and supplying water volume. Table S2. The startup flow profile within 4 $\dot{\gamma }$ ramps. Figure S1. CNF emulsion pictures of (a) the mixture of oil-water before homogenization, (b) CNFX%-DDAB0.1% and (c) CNFX%-DDAB0.5%. Figure S2. The appearance of CNF emulsion of (a) CNFX%-DDAB0.1% and (b) CNFX%-DDAB0.5%. Figure S3. The actual photo and its sectional view of liquid cell that used to load CNF emulsion. Figure S4. The dielectric raw data of 0.1 wt.% DDAB emulsion of (a,b) CNF1%, (c,d) CNF1.5%, (e,f) CNF2% and (g,h) CNF2.5% at all time intervals with the real and imaginary parts of permittivity and loss angle on dependence of dielectric frequency. Figure S5. The dielectric raw data of 0.1 wt.% DDAB emulsion of (a,b) GO1% and (c,d) GO1.5% at all time intervals with the real and imaginary parts of permittivity and loss angle on dependence of dielectric frequency so as to compare the common properties of CNF emulsion. Figure S6. The pictures of (a) before and (b) after dielectric tests of CNF emulsion relating to carbonization. Figure S7. The original data of startup-flow for 2 typical CNF emulsions. 1–4 ramps of shear rate were plotted with stress and normal force but the first ramp did not behave well because previous large deformations do not largely change the structure. Figure S8. (a) Zeta-potential raw data for a sample with DDAB/CNF-ratio of 0. (b) Zeta-potential vs. DDAB/CNF-ratio

## References

[1] Siqueira G., Kokkinis D., Libanori R., Hausmann M.K., Gladman A.S., Neels A., Tingaut P., Zimmermann T., Lewis J.A., Studart A.R. (2017). Cellulose nanocrystal inks for 3d printing of textured cellular architectures. Adv. Funct. Mater..

[2] Habibi Y., Lucia L.A., Rojas O.J. (2010). Cellulose nanocrystals: Chemistry, self-assembly, and applications. Chem. Rev..

[3] Bai W., Holbery J., Li K. (2009). A technique for production of nanocrystalline cellulose with a narrow size distribution. Cellulose.

[4] Rampinelli G., Di Landro L., Fujii T. (2009). Characterization of biomaterials based on microfibrillated cellulose with different modifications. J. Reinf. Plast. Compos..

[5] Syverud K., Stenius P. (2008). Strength and barrier properties of MFC films. Cellulose.

[6] Iwatake A., Nogi M., Yano H. (2008). Cellulose nanofiber-reinforced polylactic acid. Compos. Sci. Technol..

[7] Wang J., Tavakoli J., Tang Y. (2019). Bacterial cellulose production, properties and applications with different culture methods—A review. Carbohydr Polym.

[8] Halib N., Ahmad I., Grassi M., Grassi G. (2019). The remarkable three-dimensional network structure of bacterial cellulose for tissue engineering applications. Int. J. Pharm..

[9] Picheth G.F., Pirich C.L., Sierakowski M.R., Woehl M.A., Sakakibara C.N., de Souza C.F., Martin A.A., da Silva R., de Freitas R.A. (2017). Bacterial cellulose in biomedical applications: A review. Int. J. Biol. Macromol..

[10] Mohammadkazemi F., Azin M., Ashori A. (2015). Production of bacterial cellulose using different carbon sources and culture media. Carbohydr. Polym..

[11] Li Q., Wang Y.X., Wu Y.H., He K.H., Li Y., Luo X.G., Li B., Wang C.T., Liu S.L. (2019). Flexible cellulose nanofibrils as novel pickering stabilizers: The emulsifying property and packing behavior. Food Hydrocoll..

[12] Capron I., Cathala B. (2013). Surfactant-free high internal phase emulsions stabilized by cellulose nanocrystals. Biomacromolecules.

[13] Liu X., Shi S., Li Y., Forth J., Wang D., Russell T.P. (2017). Liquid tubule formation and stabilization using cellulose nanocrystal surfactants. Angew. Chem. Int. Ed. Engl..

[14] Salas C., Nypelö T., Rodriguez-Abreu C., Carrillo C., Rojas O.J. (2014). Nanocellulose properties and applications in colloids and interfaces. Curr. Opin. Colloid Interface Sci..

[15] Xu X., Liu F., Jiang L., Zhu J.Y., Haagenson D., Wiesenborn D.P. (2013). Cellulose nanocrystals vs. cellulose nanofibrils: A comparative study on their microstructures and effects as polymer reinforcing agents. ACS Appl. Mater. Interfaces.

[16] Li M.C., Wu Q., Moon R.J., Hubbe M.A., Bortner M.J. (2021). Rheological aspects of cellulose nanomaterials: Governing factors and emerging applications. Adv. Mater..

[17] Du L., Zhong T., Wolcott M.P., Zhang Y., Qi C., Zhao B., Wang J., Yu Z. (2018). Dispersing and stabilizing cellulose nanoparticles in acrylic resin dispersions with unreduced transparency and changed rheological property. Cellulose.

[18] Li M.-C., Wu Q., Song K., Lee S., Qing Y., Wu Y. (2015). Cellulose nanoparticles: Structure–morphology–rheology relationships. ACS Sustain. Chem. Eng..

[19] Kalashnikova I., Bizot H., Bertoncini P., Cathala B., Capron I. (2013). Cellulosic nanorods of various aspect ratios for oil in water Pickering emulsions. Soft Matter.

[20] Nomena E.M., van der Vaart M., Voudouris P., Velikov K.P. (2021). Rheology of oil-in-water emulsions stabilised by native cellulose microfibrils in primary plant cells dispersions. Food Struct..

[21] Tan Y., Liu Y., Chen W., Liu Y., Wang Q., Li J., Yu H. (2016). Homogeneous dispersion of cellulose nanofibers in waterborne acrylic coatings with improved properties and unreduced transparency. ACS Sustain. Chem. Eng..

[22] Kalia S., Boufi S., Celli A., Kango S. (2013). Nanofibrillated cellulose: Surface modification and potential applications. Colloid Polym. Sci..

[23] Zhang Y.Y., Zhu G.M., Dong B.Q., Wang F., Tang J., Stadler F.J., Yang G.H., Hong S.X., Xing F. (2021). Interfacial jamming reinforced Pickering emulgel for arbitrary architected nanocomposite with connected nanomaterial matrix. Nat. Commun..

[24] Binks B.P. (2002). Particles as surfactants—Similarities and differences. Curr. Opin. Colloid Interface Sci..

[25] Hong J.S., Ruhs P.A., Fischer P. (2015). Localization of clay particles at the oil-water interface in the presence of surfactants. Rheol. Acta.

[26] Gelot A., Friesen W., Hamza H.A. (1984). Emulsification of oil and water in the presence of finely divided solids and surface-active agents. Colloids Surf..

[27] Binks B.P., Lumsdon S.O. (2000). Effects of oil type and aqueous phase composition on oil-water mixtures containing particles of intermediate hydrophobicity. Phys. Chem. Chem. Phys..

[28] Hong J.S., Fischer P. (2016). Bulk and interfacial rheology of emulsions stabilized with clay particles. Colloids Surf. A Physicochem. Eng. Asp..

[29] Whitby C.P., Fornasiero D., Ralston J. (2008). Effect of oil soluble surfactant in emulsions stabilised by clay particles. J. Colloid Interface Sci..

[30] Shahidi S., Koch C.R., Bhattacharjee S., Sadrzadeh M. (2017). Dielectric behavior of oil–water emulsions during phase separation probed by electrical impedance spectroscopy. Sens. Actuators B Chem..

[31] Jiang Q., Sun N., Kumar P., Li Q., Liu B., Li A., Wang W., Gao Z. (2020). Real-time analysis of the stability of oil-in-water pickering emulsion by electrochemical impedance spectroscopy. Molecules.

[32] de Oliveira H.P., de Melo C.P. (2011). b Use of electrical impedance spectroscopy as a practical method of investigating the formation of aggregates in aqueous solutions of dyes and surfactants. J. Phys. Chem. B.

[33] Ghasemi S., Darestani M.T., Abdollahi Z., Hawkett B.S., Comes V.G. (2014). Electrical impedance spectroscopy for determining critical micelle concentration of ionic emulsifiers. Colloids Surf. A Physicochem. Eng. Asp..

[34] Klein R.J., Zhang S., Dou S., Jones B.H., Colby R.H., Runt J. (2006). Modeling electrode polarization in dielectric spectroscopy: Ion mobility and mobile ion concentration of single-ion polymer electrolytes. J. Chem. Phys..

[35] Sjöblom J., Skodvin T., Jakobsen T., Dukhin S.S. (1994). Dielectric spectroscopy and emulsions. A theoretical and experimental approach. J. Dispers. Sci. Technol..

[36] Sen S., Boyd R.H. (2008). Dielectric relaxation in amorphous linear aliphatic copolyesters. Eur. Polym. J..

[37] Boyle M.H. (1985). The electrical-properties of heterogeneous mixtures containing an oriented spheroidal dispersed phase. Colloid Polym. Sci..

[38] Hill R.M., Cooper J. (1992). Characterization of water-in-oil emulsions by means of dielectric-spectroscopy. J. Mater. Sci..

[39] Skodvin T., Sjoblom J. (1996). Models for the dielectric properties of flocculated w/o-emulsions. J. Colloid Interface Sci..

[40] Gorbacheva S.N., Ilyin S.O. (2021). Morphology and rheology of heavy crude oil/water emulsions stabilized by microfibrillated cellulose. Energy Fuels.

[41] Chen J.H., Liu J.G., Su Y.Q., Xu Z.H., Li M.C., Ying R.F., Wu J.Q. (2019). Preparation and properties of microfibrillated cellulose with different carboxyethyl content. Carbohydr. Polym..

[42] Ougiya H., Watanabe K., Morinaga Y., Yoshinaga F. (1997). Emulsion-stabilizing effect of bacterial cellulose. Biosci. Biotechnol. Biochem..

[43] Winuprasith T., Suphantharika M. (2015). Properties and stability of oil-in-water emulsions stabilized by microfibrillated cellulose from mangosteen rind. Food Hydrocoll..

[44] Kalashnikova I., Bizot H., Cathala B., Capron I. (2011). New Pickering emulsions stabilized by bacterial cellulose nanocrystals. Langmuir.

[45] Lu Y., Qian X.L., Xie W.Y., Zhang W.T., Huang J., Wu D.F. (2019). Rheology of the sesame oil-in-water emulsions stabilized by cellulose nanofibers. Food Hydrocoll..

[46] Costa C., Mira I., Benjamins J.-W., Lindman B., Edlund H., Norgren M. (2019). Interfacial activity and emulsion stabilization of dissolved cellulose. J. Mol. Liq..

[47] Kim J.-H., Shim B.S., Kim H.S., Lee Y.-J., Min S.-K., Jang D., Abas Z., Kim J. (2015). Review of nanocellulose for sustainable future materials. Int. J. Precis. Eng. Manuf.-Green Technol..

[48] Dickinson E. (2003). Hydrocolloids at interfaces and the influence on the properties of dispersed systems. Food Hydrocoll..

[49] Zembyla M., Lazidis A., Murray B.S., Sarkar A. (2019). Water-in-oil pickering emulsions stabilized by synergistic particle-particle interactions. Langmuir.

[50] Costa A.L.R., Gomes A., Cangussu L.B., Cunha R.L., de Oliveira L.S., Franca A.S. (2022). Stabilization mechanisms of O/W emulsions by cellulose nanocrystals and sunflower protein. Food Res. Int..

[51] Stadler F.J., Cui S.M., Hashmi S., Handschuh-Wang S., Li W.Q., Wang S.C., Yan Z.C., Zhu G.M. (2022). Multiple interval thixotropic test (miTT)-an advanced tool for the rheological characterization of emulsions and other colloidal systems. Rheol. Acta.

[52] Matos M., Marefati A., Bordes R., Gutierrez G., Rayner M. (2017). Combined emulsifying capacity of polysaccharide particles of different size and shape. Carbohydr. Polym..

[53] Zhou H., Lv S., Liu J., Tan Y., Muriel Mundo J.L., Bai L., Rojas O.J., Mclements D.J. (2020). Modulation of physicochemical characteristics of Pickering emulsions: Utilization of nanocellulose- and nanochitin-coated lipid droplet blends. J. Agric. Food Chem..

[54] Jiang Y., Liu L.L., Wang B.J., Sui X.F., Zhong Y., Zhang L.P., Mao Z.P., Xu H. (2018). Cellulose-rich oleogels prepared with an emulsion-templated approach. Food Hydrocoll..

[55] Patel I., Woodcock J., Beams R., Stranick S.J., Nieuwendaal R., Gilman J.W., Mulenos M.R., Sayes C.M., Salari M., DeLoid G. (2021). Fluorescently labeled cellulose nanofibers for environmental health and safety studies. Nanomaterials.

[56] Pan X.D., McKinley G.H. (1997). Characteristics of electrorheological responses in an emulsion system. J. Colloid Interface Sci..

[57] Richert R., Wagner H. (1998). The dielectric modulus: Relaxation versus retardation. Solid State Ion..

[58] Valentini L., Bittolo Bon S., Cardinali M., Fortunati E., Kenny J.M. (2014). Cellulose nanocrystals thin films as gate dielectric for flexible organic field-effect transistors. Mater. Lett..

[59] Niu F., Li M., Huang Q., Zhang X., Pan W., Yang J., Li J. (2017). The characteristic and dispersion stability of nanocellulose produced by mixed acid hydrolysis and ultrasonic assistance. Carbohydr. Polym..

[60] Skodvin T., Sjoblom J., Saeten J.O., Warnheim T., Gestblom B. (1994). A time-domain dielectric-spectroscopy study of some model emulsions and liquid margarines. Colloids Surf. A Physicochem. Eng. Asp..

[61] Nomena E.M., Remijn C., Rogier F., van der Vaart M., Voudouris P., Velikov K.P. (2018). Unravelling the mechanism of stabilization and microstructure of oil-in-water emulsions by native cellulose microfibrils in primary plant cells dispersions. ACS Appl. Bio. Mater..

[62] Chen Y., Xu C., Huang J., Wu D., Lv Q. (2017). Rheological properties of nanocrystalline cellulose suspensions. Carbohydr. Polym..

[63] Jia X., Chen Y., Shi C., Ye Y., Abid M., Jabbar S., Wang P., Zeng X., Wu T. (2014). Rheological properties of an amorphous cellulose suspension. Food Hydrocoll..

[64] Yasuda K., Armstrong R.C., Cohen R.E. (1981). Shear flow properties of concentrated solutions of linear and star branched polystyrenes. Rheol. Acta.

[65] Chanamai R., Mclements D.J. (2000). Dependence of creaming and rheology of monodisperse oil-in-water emulsions on droplet size and concentration. Colloids Surf. A Physicochem. Eng. Asp..

