# Supplementary material for: Influence of Cellulose Nanofibers on the Behavior of Pickering Emulsions. Part 1. Microscopy and Startup Flow Test
Source: Materials (Basel). 2022 Nov 22;15(23):8285. doi: 10.3390/ma15238285 (PMC9736908; doi:10.3390/ma15238285)
Supplement: Supplementary file 1 [file materials-15-08285-s001.zip › materials-1993625-supplementary.pdf]

materials

Supplementary information

Influence of Cellulose Nanofibers on the Behavior of Pickering Emulsions. Part 1. Microscopy and Startup Flow Test

Shuming Cui[1,2], Saud Hashmi[3], Wenqiang Li[1], Stephan Handschuh-Wang[4], Chengtian Zhu[1], Shichang Wang[1], Yan-Fei Huang[1*], Guangming Zhu[1*] and Florian J. Stadler[1*].

Sample preparation

Figure S1a,b show the pictures of all CNF emulsion samples after mixing of oil and aqueous phase and homogenization. The volume of CNF aqueous dispersion (downside the bottle) and mixture oil phase (upside) have nearly height difference with the ratio of 2:1. It is obvious that the increase amount of CNF dispersion (white floc) formed higher concentration and viscosity of CNF emulsion (Figure S1a). With 4.5 ± 0.5 wt.% CNF aqueous dispersion as the raw nanofibrillated cellulose material, all the CNF dispersion need to be diluted by distilled water, which listed at Table S1 for ca.2 mL aqueous phase and 1mL mixture oil. For instance, CNF1% means that 2 mL aqueous phase is composed of 0.467 g raw CNF gel and 1.556 mL water supply. The amount of supplying water is followed by the Equation (S1).

$$V_{supplying\ water} = 2 - \frac{2C_{CNF}}{4.5}\ (\text{mL}), m_{CNF} = \frac{2\rho C_{CNF}}{4.5}\ (g) \qquad \text{(S1)}$$

Here, C_{CNF} is the CNF dispersion in real concentration and m_{CNF} is the obtained weight of gel-like CNF, while the density of raw CNF gel is about 1.05 g/mL. The CNF emulsion after homogenization were captured and shown at Figure S1b for 0.1wt% DDAB and Figure S1c for 0.5wt% DDAB with the inverted placement that observed the yield behavior. The emulsions of CNF0.5%-DDAB0.1%, CNF0.5%-DDAB0.5%, and CNF1%-DDAB0.1% have too low viscosity to totally flow down at inverted bottle because of the weak yield stress, while CNF1.25&1.5%-DDAB0.1% have partly falling down. The strong and entire adhesive happens in emulsion of larger than 1.5 wt.% CNF. Due to the promoted adsorption between CNF and surface by means of DDAB, DDAB0.5%-series are more viscous gel-like behavior compared with DDAB0.1%-series and do not present obvious phase separation except of CNF0.5%-DDAB0.5% (Figure S1c, S2a,b).

Table S1. The component ratio of aqueous phase including CNF gel weight and supplying water volume.

Aqueous phase (2 mL)	Raw 4.5 wt% CNF gel (g)	Water supply (mL)
CNF0.5%	0.233	1.778
CNF1%	0.467	1.556
CNF1.25%	0.583	1.444
CNF1.5%	0.700	1.333
CNF1.75%	0.817	1.222
CNF2%	0.933	1.111
CNF2.5%	1.167	0.889
CNF2.75%	1.283	0.778
CNF3%	1.400	0.667

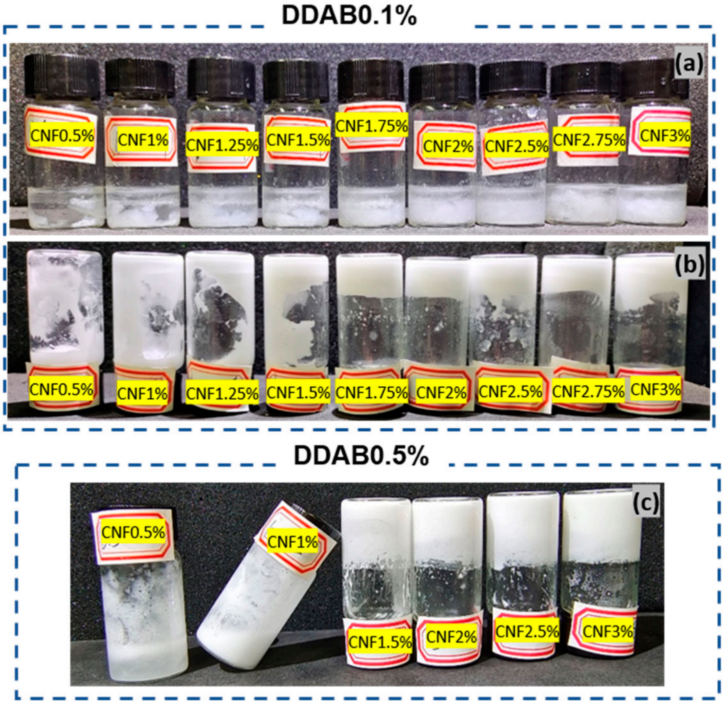

Figure S1. CNF emulsion pictures of (a) the mixture of oil-water before homogenization, (b) CNFX%-DDAB0.1% and (c) CNFX%-DDAB0.5%.

Figure S2a,b show the direct appearance of CNF emulsions and observe that gel-like emulsion are formed at higher CNF and DDAB content. For higher CNF content, the dense polymer chains become an elastic and crosslinked network to support droplets, while DDAB enhance the adsorption between CNF and surface and promote the formation of CNF clusters in aqueous phase to obtain temporary solid-type, because merely the τ_0 and η_0 increase at initial deformation, domain (low $\dot{\gamma}$) with higher DDAB (Figure 10). However, an interesting discovery is that CNF0.5%-DDAB0.5% has more bubbles than CNF0.5%-DDAB0.1%.

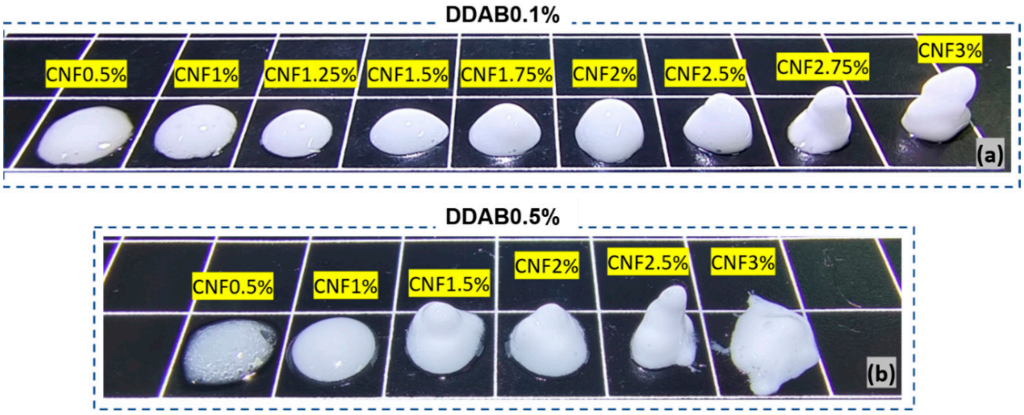

Figure S2. The appearance of CNF emulsion of (a) CNFX%-DDAB0.1% and (b) CNFX%-DDAB0.5%.

Samples and basic theory of EDS

Figure S3 showed the sample cell and its various components using for dielectric test. In general, liquid or flow -like sample can be measured by placing them between two open disposable electrodes, which are loaded in the carrier owing to prevent water evaporation and leakage. The carrier includes upper closing plate, lower carrier plate, and Teflon isolation that is a white color ring and used to prevent water evaporation. What's more, the

electrodes' area are filled totally with emulsion in order to prevent the potential influences from outside electric fields. Importantly, two parallel additional spacers that are made of 0.1mm rod-like Silica with low tan δ, constant permittivity, and resistance over the used temperature and frequency range need to be installed in electrode spacing to stabilize the gap. For the conductive units, the upper electrode is pressed by a spring and the cell closing plate. The two seal rings attached to the Teflon isolation (white color ring on the actual cell appearance) for preventing evaporation of sample.

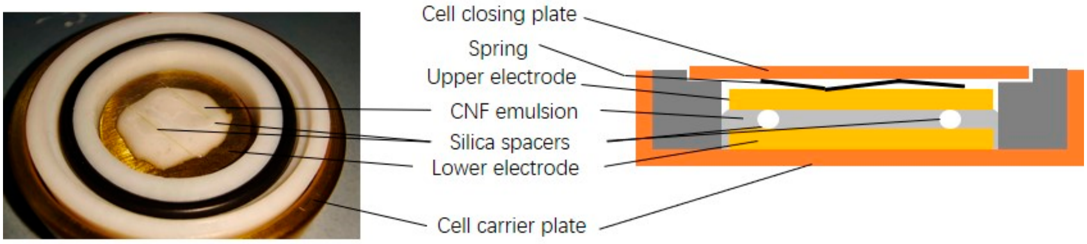

Figure S3. Photo and its schematic sectional view of liquid cell that used to load CNF emulsion.

The electric signals of input absolute AC voltage U^* and output absolute current I^* can be measured by EDS and then figure out several dielectric parameters, such as complex impedance z^*, complex conductivity σ^*, complex permittivity ε^*, and complex capacity $\boldsymbol{c}^*$. The reason is that the hysteresis that defined as phase angle φ are always existed between U^* and I^* when the dielectric medium were loaded, which can be expressed in Equation (S2).

$$U^*(\omega t) = U'(\omega t) + iU''(\omega t), \text{ while } I^*(\omega t + \varphi) = I'(\omega t + \varphi) + iI''(\omega t + \varphi) \quad \text{(S2)}$$

Here, ω is the value related to the frequency: $\omega = 2\pi f$ and U', U'', I', and I'' are the real and imaginary parts of AC voltage and current, respectively. The complex impedance z^* is defined as the ratio of U^* and I^* and deduce to the complex conductivity σ^*, defined in Equations (S3) and (S4):

$$z^* = z' + iz'' = \frac{U^*}{I^*} = \frac{\sigma^*}{i\omega C_0 \varepsilon_0} \quad \text{(S3)}$$

$$C_0 = \frac{\pi}{4}\frac{D^2}{d}\varepsilon_0 \quad \text{S4)}$$

Here, ε_0=8.859 pFm^{-1} is the vacuum permittivity, which is lower than any material permittivity. C_0 is the empty cell capacity and calculated by the area of the parallel electrode and the thickness of loaded samples. z^* is subjected to the intrinsic properties of CNF emulsion rather than the AC voltage or current. Moreover, σ^* and c^* have their relation with ε^* following the Equations (S5) and (S6).

$$\sigma^* = \sigma' + i\sigma'' = i\omega\varepsilon_0\varepsilon^* \quad \text{(S5)}$$

$$\varepsilon^* = \varepsilon' + i\varepsilon'' = \frac{C^*}{C_0} \quad \text{S6)}$$

Here, c^* is the complex sample capacity and is usually affected by external capacity, which can be solved by adjusting the electrodes to minimum *d*/*D*. From these equations, there is a significant proportionality among σ^*, ε^*, z^*, and c^*. Furthermore, ε' is used to calculate the polarization *P* in case of known electric current density *D* and field intensity *E*, which is expressed by Equation (S7):

$$D = \varepsilon'(\omega)\varepsilon_0 E = (1 + \chi)\varepsilon_0 E = \varepsilon_0 E + P \quad \text{(S7)}$$

Here, χ is the susceptibility. The polarization process of CNF emulsion is divided into three kinds: electron and ion movement and the orientation of dipole moment. In CNF emulsions, the time scale of the interfacial polarization is dominated by orientational molecular mobility as the main process, which affects by the behavior of CNF film at the AC field. The process of the first two polarization is transient, thus, orientation polarization is mainly discussed in this paper. When it comes to the permittivity dependence on frequency, ε' and ε'' show typical decrease trends with higher frequency in accordance with the Debye's classical equations (Equation (S8)). Their phase differences between real and imaginary part of permittivity were calculated by Equation (S9), which is different concept with phase angle φ that is defined phase differences of current or voltage:

$$\varepsilon' = \varepsilon_\infty + \frac{\varepsilon_S-\varepsilon_\infty}{1+\omega^2\tau^2} \ \& \ \varepsilon'' = \frac{(\varepsilon_S-\varepsilon_\infty)\omega\tau}{1+\omega^2\tau^2} \quad \text{(S8)}$$

$$\delta = \arctan\left[\frac{\varepsilon''(\omega)}{\varepsilon'(\omega)}\right] = \frac{\varepsilon_s-\varepsilon_\infty}{\varepsilon_s+\varepsilon_\infty} \quad \text{(S9)}$$

Equation (S8) reveals that the permittivity of dielectric medium is $\varepsilon_s = \varepsilon'$ in the case of DC field ($f = \omega = 0$), but show the $\varepsilon' = \varepsilon_\infty$ in the case of optical frequency ($f \to \infty$).

The AC electric field stimulates the sample for several periods of 720s time increment and every intervals data for CNF 1-2.5 %-DDAB0.1% are displayed in Figure S4 with permittivity and loss angle δ on dependence of frequency. In general, the first two intervals show the different curves shape in comparison with other intervals and the last two intervals maintain similar curve regulation. After three times of frequency sweep, the lowest δ move upside and ε'' increase totally, then they would concentrate to the equilibrium response at same time the overlapping curves arise. CNF2.5%-DDAB0.1% have nearly overlapped curves at the last two intervals, but such situation cannot be searched with the emulsion at lower CNF content, which means it needs to take a longer test time. Another investigation that also can be found between the curves of Figure S4a,c,e,g and b,d,f,h) is the closer overlapping curve at real and imaginary parts of permittivity than δ-plot. Thus, the plotting of δ on dependence of frequency can illustrate obviously the contrast about the dielectric properties by varying CNF or DDAB content. In case of better comparison of any samples, the data of last intervals would tend to balance and get more precise results.

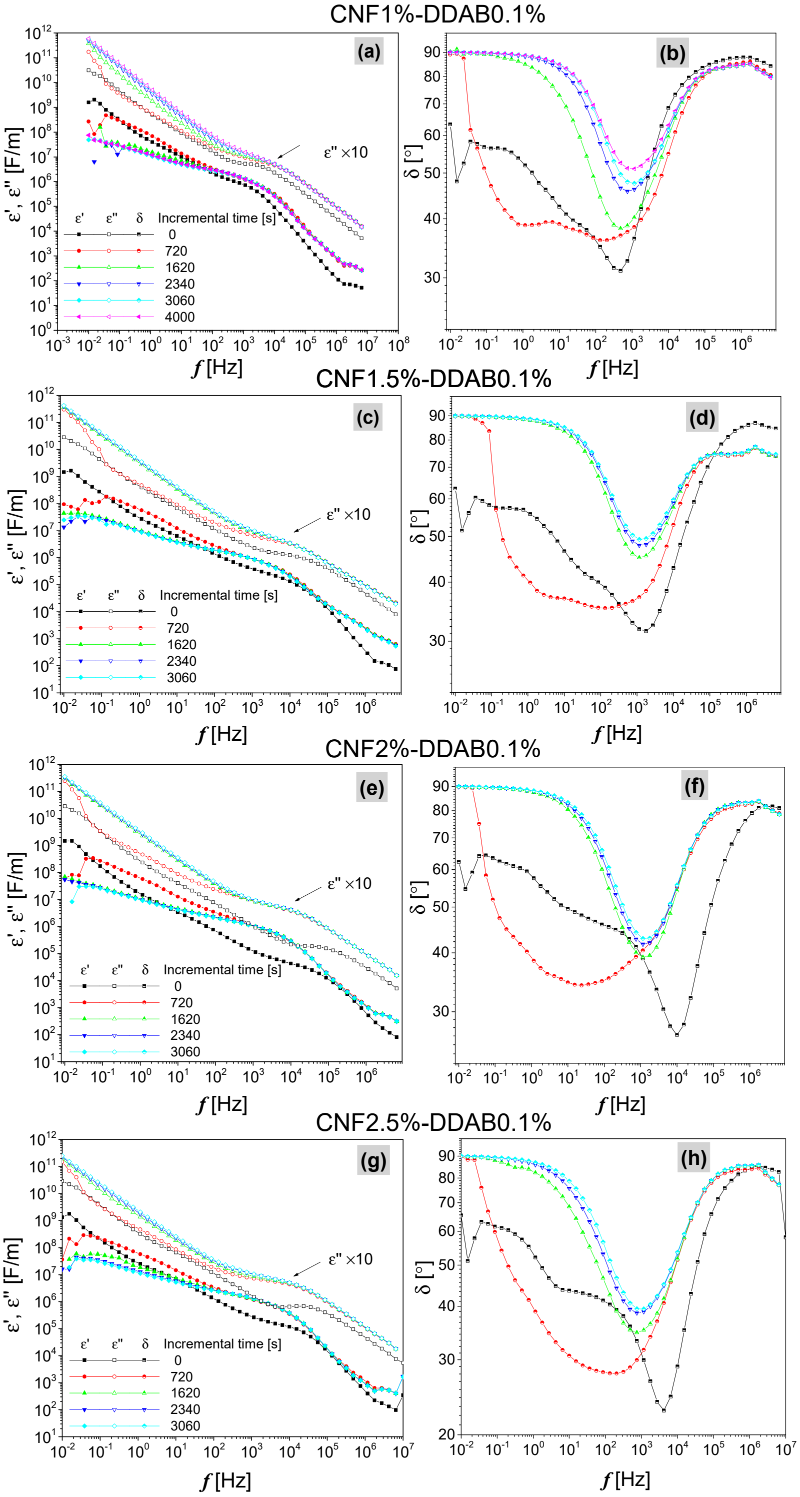

Figure S4. The dielectric raw data including all intervals: function of $\varepsilon'(f)$, $\varepsilon''(f)$, and $\delta(f)$ for CNF-series: (a,b) 1%, (c-d) 1.5%, (e-f) 2%, and (g-h) 2.5% with 0.1wt% DDAB emulsions, respectively.

According to "$\delta \sim f$"-plot in Figure S4b,d,f,g, δ show commonly single minimum value at same frequency value after several test loop, which show a gradual tendency of constant state and prove that CNF, as a unitary value play a decisive role in hysteresis. To prove the inference, the same dielectric profile of another emulsions, GO Pickering emulsions with same oil and DDAB component, have common minimum δ at higher frequency (Figure S5). Moreover, ε″ of GO emulsion do not behave the slight plateau at around f=10^3 Hz, but at f=10 Hz. Both of two differences can significantly prove the

unique dielectric properties, such as modulus, permittivity, conductivity, and impedance of CNF stabilized emulsions.

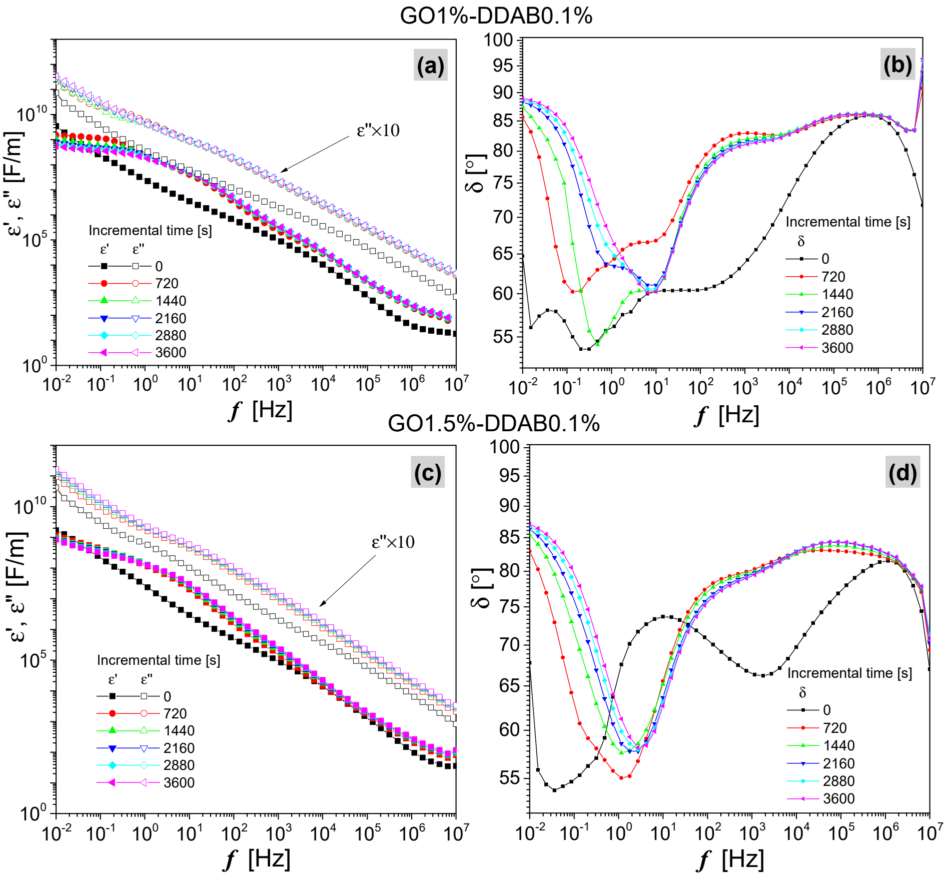

Figure S5. The dielectric raw data including all intervals: function of $\varepsilon'(f)$, $\varepsilon''(f)$, and $\delta(f)$ for (a, b) GO1%-DDAB0.1% and (c, d) GO1.5%-DDAB0.1%, respectively.

In order to illustrate the tendency of $\varepsilon'(f)$, $\varepsilon''(f)$, and $\delta(f)$ during 5 loops of frequency domain, the emulsion's appearances of before or after test are shown in Figure S6. The initial appearance of one CNF2.5%-DDAB0.1% emulsion is shown in Figure S6a for comparison with the appearance after totally 5 frequency ramps (Figure S6b). The changing of "white to dark" means that the emulsion carbonization associate from voltage damage and illustrates the formation of stable conduction matrix and proper gap.

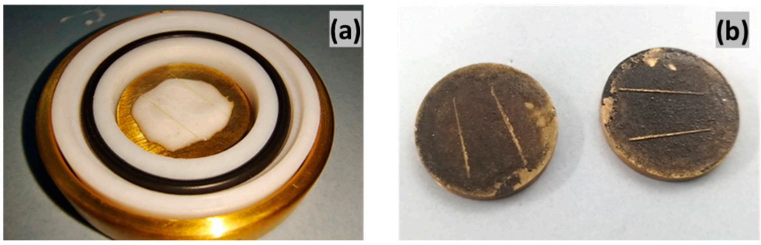

Figure S6. The pictures of (a) before and (b) after dielectric tests of CNF emulsion.

Startup flow profile

Table S2 show the startup flow profile with exponentially increasing or decreasing $\dot{\gamma}$ and a ramp time of 900 s (in total 3600 s for 4 ramps). Such setting is performed to check to obtain a comparison with the results of the SUF intervals of the miTT protocol.

Table S2. The startup flow profile within 4 shear rate ramps.

**Interval**	**1**	**2**	**3**	**4**
Point	460	460	460	460
$\dot{\gamma}$ (1/s)	0.01→1000	0.01←1000	0.01→1000	0.01←1000

Figure S7 show the $\tau(\dot{\gamma})$ and $F_N(\dot{\gamma})$ raw data of startup-flow test for two different emulsions. The typical original flow behavior is shown for the first ramp that is obvious unstable data of stress and normal force because the bulk movement of droplets need to be drove. That means the data of first ramp can be ignore and the last ramp data need to be selected due to obtain the more stable curves. Moreover, the similar data for 2-4 ramps represent the continuous deformation of droplets, which illustrates the constant flow behavior for these CNF emulsions. More importantly, the effects associating with DDAB absorption is critically strong owing to the "stress step" appearing among 2-4 ramps.

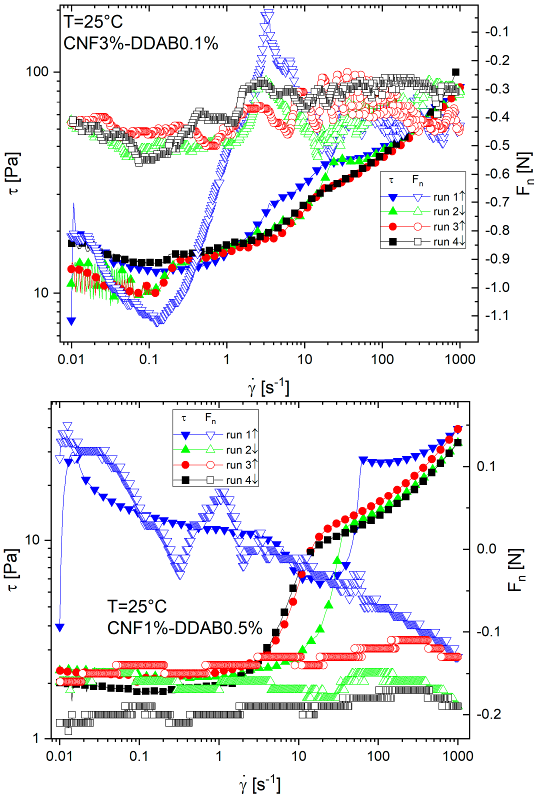

Figure S7. The original data of startup flow for 2 typical CNF emulsions. 1-4 ramps of shear rate were plotted with stress and normal force but the first ramp did not behave well because previous large deformations do not largely change the structure.

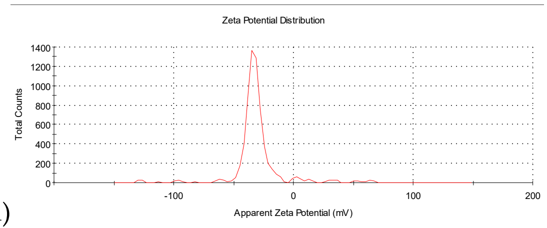

(a)

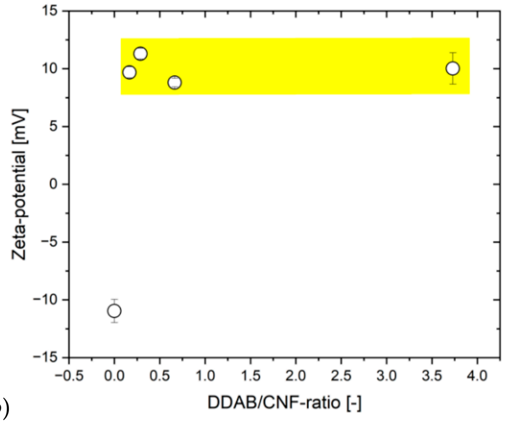

(**b**)

Figure S8. (**a**) Zeta-potential raw data for a sample with DDAB/CNF-ratio of 0. (**b**) Zeta-potential vs. DDAB/CNF-ratio.
